# Physician perspectives on integration of artificial intelligence into diagnostic pathology

**DOI:** 10.1038/s41746-019-0106-0

**Published:** 2019-04-26

**Authors:** Shihab Sarwar, Anglin Dent, Kevin Faust, Maxime Richer, Ugljesa Djuric, Randy Van Ommeren, Phedias Diamandis

**Affiliations:** 1 Department of Laboratory Medicine and Pathobiology, University of Toronto, Toronto, Ontario M5S 1A8 Canada; 2 Department of Computer Science, University of Toronto, 40 St. George Street, Toronto, Ontario M5S 2E4 Canada; 3 Princess Margaret Cancer Centre, MacFeeters Hamilton Centre for Neuro-Oncology Research, 101 College Street, Toronto, Ontario M5G 1L7 Canada; 4 Laboratory Medicine Program, Department of Pathology, University Health Network, 200 Elizabeth Street, Toronto, ON M5G 2C4 Canada

**Keywords:** Health care, Health occupations

## Abstract

Advancements in computer vision and artificial intelligence (AI) carry the potential to make significant contributions to health care, particularly in diagnostic specialties such as radiology and pathology. The impact of these technologies on physician stakeholders is the subject of significant speculation. There is however a dearth of information regarding the opinions, enthusiasm, and concerns of the pathology community at large. Here, we report results from a survey of 487 pathologist-respondents practicing in 54 countries, conducted to examine perspectives on AI implementation in clinical practice. Despite limitations, including difficulty with quantifying response bias and verifying identity of respondents to this anonymous and voluntary survey, several interesting findings were uncovered. Overall, respondents carried generally positive attitudes towards AI, with nearly 75% reporting interest or excitement in AI as a diagnostic tool to facilitate improvements in workflow efficiency and quality assurance in pathology. Importantly, even within the more optimistic cohort, a significant number of respondents endorsed concerns about AI, including the potential for job displacement and replacement. Overall, around 80% of respondents predicted the introduction of AI technology in the pathology laboratory within the coming decade. Attempts to identify statistically significant demographic characteristics (e.g., age, sex, type/place of practice) predictive of attitudes towards AI using Kolmogorov–Smirnov (KS) testing revealed several associations. Important themes which were commented on by respondents included the need for increasing efforts towards physician training and resolving medical-legal implications prior to the generalized implementation of AI in pathology.

## Introduction

Over the course of several decades, the use of computational approaches for medical applications has increased steadily. In specialities which focus on image analysis for medical diagnostics such as radiology and pathology, considerable effort has been put towards the development of algorithms that can assist the clinician in image interpretation for diagnosis, prognostication, and production of clinical reports.^1^ Advances in machine learning approaches have given rise to powerful learning algorithms, collectively termed artificial intelligence (AI). A subset of AI platforms using deep learning approaches have developed powerful capabilities in specific domains as demonstrated by achievements in the games of chess, shogi, and Go, amongst other.^2,3^ The success of deep learning in these domains has stimulated intense interest in the development and application of intelligent systems for real-world applications in many fields, including health care. In recent years, various groups have published reports of deep learning applications for fields such as medicine,^4^ radiology,^5–8^ dermatology^9,10^ ophthalmology,^11^ and pathology.^12–23^ In diagnostic fields such as radiology and pathology, AI carries the potential to transform the clinical practice of physicians. In pathology specifically, AI-based diagnostic platforms may perform image analysis for tissue histology, analyse molecular outputs from diagnostic tests such as next-generation sequencing (NGS), and integrate these with clinical and/or radiological characteristics to improve the predictive and prognostic power of traditional pathology approaches.^24,25^

The perspectives of practising clinicians and diagnosticians on the integration of AI into medical practice are poorly understood. This is especially true in pathology, where only recently has a whole slide imaging system produced by Philips been approved by the U.S. Food and Drug Administration (FDA).^26^ Given the possibility that AI systems will become integrated into diagnostic pathology, we felt it was timely and important to begin evaluating and understanding the attitudes, opinions, and feelings of practising pathologists and trainees around this technology. Differences in physician perspectives across specialties and other demographic parameters could directly impact the rate and effectiveness of implementation, and better clarifying these will help to ensure the priorities of all stakeholders are considered. Specifically, pathologists’ concerns about impacts on job security, ability to adapt to novel technologies, and impact on clinical and research careers can be more effectively addressed by hospitals and pathology departments if they are well understood. To this end, we performed an online survey of pathologist colleagues on topics regarding incorporation of AI into clinical practice, its impact on research, and pathologists’ projections for the future of pathology training and teaching.

## Results

### Respondent *d*emographics

**Fig. 1:**
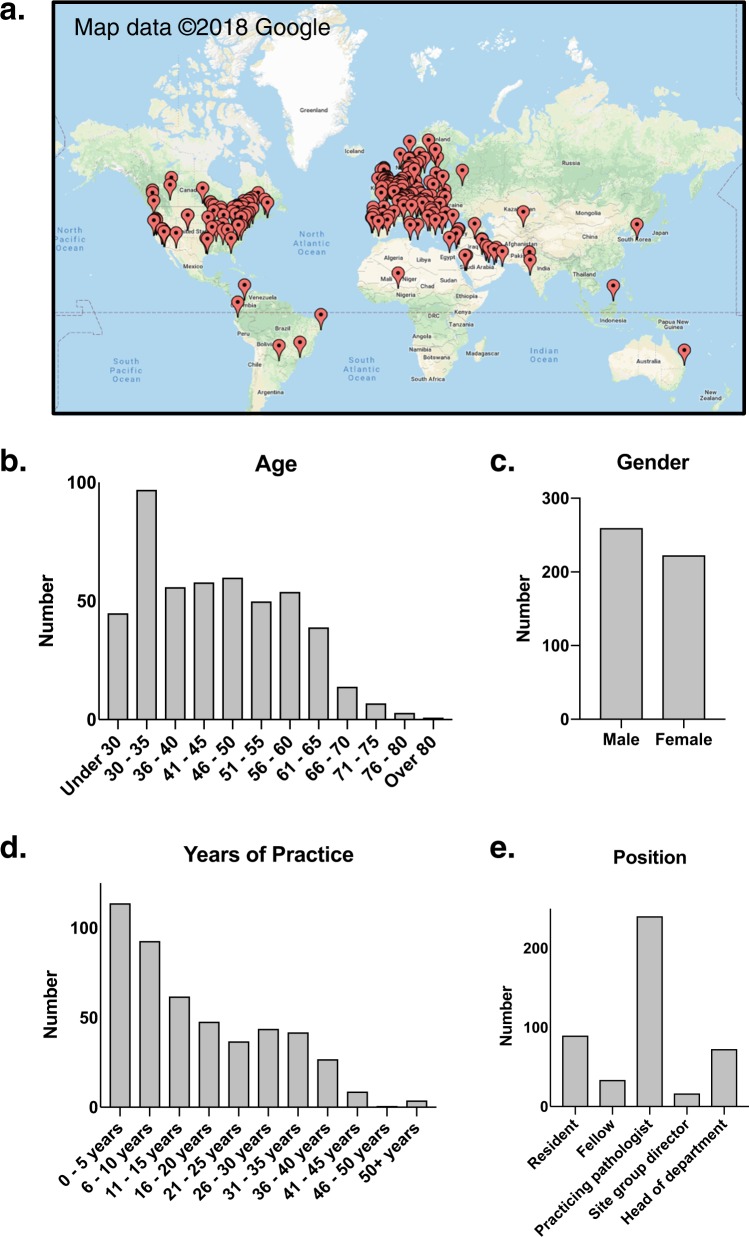
Demographic data of survey respondents. **a** City of pathology practice/training of all survey respondents (473 responses, 14 skipped), (Maps data ©2018 Google). **b** Age distribution of survey respondents (484 responses, 3 skipped). **c** Gender demographic of respondents (484 responses, 3 skipped). **d** Years of pathology practice (including training) of survey respondents (481 responses, 6 skipped). **e** Reported positions held by survey respondents. Some of the responses within “Other” included positions that could be grouped within “Practicing Pathologist”, but are presented separately as described by respondents (486 responses, 1 skipped)

**Fig. 2:**
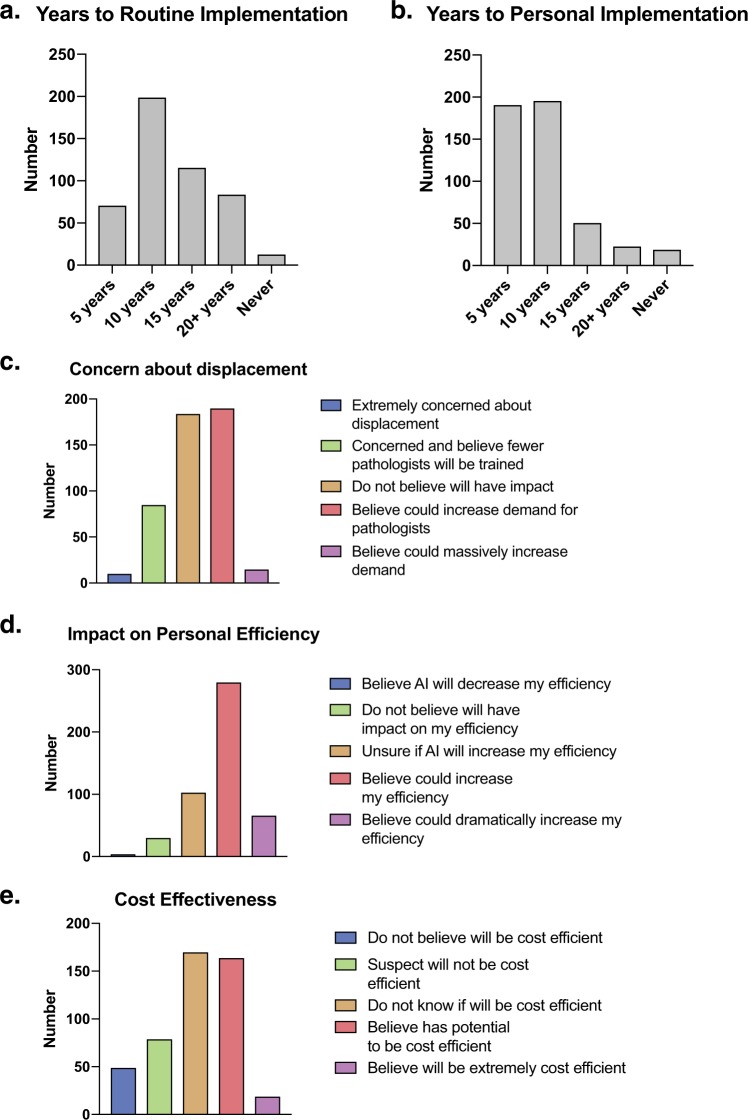
Opinions on the use of AI in clinical pathology practice. **a** Predicted interval to implementation in personal practice (480 responses, 7 skipped). **b** Predicted interval to implementation in routine practice (480 responses, 7 skipped). **c** Level of concern that pathologists will be displaced by AI tools (484 responses, 3 skipped). **d** Pathologists’ perspectives on impact of AI tools on personal efficiency (483 responses, 4 skipped). **e** Pathologists’ perspectives on cost-effectiveness of AI tools implementation (481 responses, 6 skipped)

Survey responses were collected from a total of 487 respondents in 59 countries (Fig. 1a). The majority of respondents practiced in Canada (119, 24.9%), USA (106, 22.2%), and the UK (50, 10.5%). Respondent age was spread over a wide range of age groups, with most frequent age group being 30–35 (97, 20.0%), and relatively smaller numbers below the age of 30 (45, 9.30%) and over the age of 66 (25, 5.2%) (Fig. 1b). We received a slightly increased number of male respondents (260, 53.7%) compared to female respondents (223, 46.1%). 23.7% of respondents reported having practiced less than 5 years, 19.3% between 6 and 10 years, and relatively fewer respondents had practiced for longer periods of time (Fig. 1d). Most respondents were practicing pathologists (241, 49.6%) or residents/fellows (124, 25.5%). While a minority of respondents answered “Other” (31, 6.4%), some of the responses included positions that could be grouped with “practicing pathologist”. Most respondent specialties were reported as general pathology, anatomic pathology, neuropathology, or multiple sites/other. Most were academic pathologists (342, 70.5%), relatively fewer were community pathologists (103, 21.2%), and the remaining practiced within other settings including government, military, or industry. Respondents were overall more likely to come from larger practices, with most coming from groups with >25 members (148, 30.5%), and relatively fewer from smaller practices with only 29 (6.0%) from groups of 21–25, 52 (10.7%) from groups of 16–20, 83 (17.1%) from groups of 11–15, 100 (20.6%) from groups of 5–10, and 67 (13.8%) from groups of less than 5. Respondent results are summarized below, and select graphical depictions can be found in Fig. 2.

### General attitudes and perspectives

Overall, respondent attitudes towards AI in diagnostic pathology were positive, with many either expressing interest in (119, 41.2%), or excitement about (155, 32.1%) the integration of AI-tools. Overall, concerns about displacement and negative career impacts were limited; many felt AI would not impact employability (184, 38.0%) or would create new positions and increase employment prospects (205, 42.4%). A smaller number reported being concerned (85, 17.6%) or extremely concerned (10, 2.1%) that AI-tools would displace human jobs. Most respondents did not feel AI-tools would impact compensation (317, 65.6%), though a minority felt compensation would be negatively impacted (80, 16.6%). Overall, many respondents predicted integration of AI into diagnostic workflow within the next five years (191, 39.8%) or ten years (196, 40.8%), with the remaining predicting implementation within 15 years (51, 10.6%) or greater than 20 years (23, 4.8%). Regarding impacts of AI-tool implementation on relationships with colleagues, most respondents either felt that use of AI-tools would not impact the way they were viewed by colleagues (210, 43.6%), or that their adoption of new technology would be welcomed (197, 40.9%). A minority felt that implementation would have a negative impact on the way they were viewed by colleagues (75, 15.6%). Regarding feelings about patient perspectives, most believed patients would have no opinion (246, 51.0%) or would be excited about its use (140, 29.1%).

### Perspectives on clinical implications of AI-tool use

Many respondents felt that with appropriate training, AI tools could increase (280, 58.0%) or even dramatically increase (66, 13.7%) diagnostic efficiency. A relatively smaller cohort felt efficiency would not be impacted (30, 6.2%), would be negatively impacted (4, 0.8%), or were unsure of impact on efficiency (103, 21.3%). Despite these positive attitudes towards AI tools, most respondents still felt that diagnostic decision making should remain a predominantly human task (231, 48.3%), or shared equally with an AI algorithm (121, 25.3%), while a smaller percentage felt that AI-tools should take a dominant role (97, 20.3%). Similarly, regarding the possibility of machine error, many respondents acknowledged concerns about the possibility of unpredictable results or artefact-related errors. Relatively few respondents did not feel concerned about AI-tool errors (119, 24.8%), or believed AI error rates would be lower compared to humans (48, 10.0%). Overall, a majority of pathologists felt that the use of AI-tools combined with human inputs for generation of diagnostic reports would help to decrease the rates of reporting errors (256, 53.0%). When asked about the role of AI-tools in quality assurance (QA) initiatives at their institutions, many felt AI would provide an additional level of QA (330, 68.6%), while relatively fewer felt AI-tools would have no impact on QA (120, 25.0%). Finally, in terms of medico-legal responsibility for diagnostic errors made by a human/AI combination, opinions ranged considerably, with 209 (43.7%) believing the platform vendor and pathologist should be held equally liable, 240 (50.2%) believing responsibility remained primarily that of the human, and finally 29 (6.1%) agreeing that the platform vendor should primarily be liable. These relatively split opinions suggest this still needs to be authoritatively resolved before the introduction of these tools into clinical practice.

### Perspectives on impacts of AI-tools on research and trainee development

A large percentage of respondents were supportive of implementation of AI into their practices if it resulted in an increase in time spent on academic or research pursuits (448, 93.3%), compared to a minority who were not (11, 2.3%), or were unsure/other response (21, 4.4%). Nearly half of respondents anticipate that the implementation of AI will permit increased research productivity and allow pathologists to answer questions that were previously not possible (259, 53.6%), while a smaller number felt there would be no impact (99, 20.5%) or a negative impact (6, 1.24%). With respect to impacts on trainee education, many respondents felt training duration would decrease (30, 6.24%), while many others felt training duration would increase (49, 10.2%), or with the addition of training in informatics during residency/fellowship (344, 71.5%). A significant number felt that they would need to dedicate more time to training residents given an expanded tool set (119, 24.7%), with a smaller number agreeing that teaching responsibilities would decrease due to improved teaching efficiency (47, 9.8%). Interestingly, with regards to impact on clinical skills or trainees and practitioners, responses were mixed, with some respondents expressing concern that AI tools would erode pathologists’ skill (126, 26.1%), while others felt AI tools would enhance development of ‘traditional skills’ (101, 21.0%), or would not affect clinical skills (164, 34.0%).

### Perspectives on implementation of AI-tools

Views on the setting in which AI-tools will be used varied, with some believing AI will be used primarily in academic settings (199, 41.3%), while the remaining majority believing usage would be similar in community and academic practices (174, 36.1%). With regards to ease of uptake and implementation, many respondents felt that AI tools will be relatively intuitive with little need for training (109, 22.7%), while others felt training from a platform representative would be of help (202, 41.1%), or a dedicated course/workshop would be necessary (139, 29.0%). This is a relatively important finding among the overwhelmingly positive respondents and signals a need for more educational resources and conferences for physician education. Relatively few believed lack of knowledge regarding AI would a pose significant difficulty (25, 5.2%), or would be an absolute barrier to implementation (5, 1.0%). In terms of preparation, many respondents had exposure to some research in the area, with 123 (25.6%) having attended/read 1-2 talks/papers on the subject, 3–5 (101, 21.0%), 5–10 (73, 15.2%), or rarely greater than 25 (45, 9.4%).

### Statistically significant associations

Application of Kolmogorov–Smirnov (KS) testing revealed a number of statistically significant associations between respondent demographic characteristics and perspectives on application of AI tools in pathology. Our testing demonstrated that compared to females, male respondents carry a more optimistic outlook on integration of AI into practice (*D* = 0.26, *n* = 259, *p* < 0.001), and are more likely to adopt before formal validation/support attained (*D* = 0.27, *n* = 221, *p* < 0.001). This interest amongst male respondents could be correlated with their reported expectation that AI could improve cost-effectiveness (*D* = 0.12, *n* = 221, *p* < 0.01), personal efficiency (*D* = 0.11, *n* = 221, *p* < 0.01), and quality assurance (*D* = 0.17, *n* = 164, *p* < 0.001). Males also reported subjectively feeling more comfortable with adopting the technology (*D* = 0.15, *n* = 219, *p* < 0.001); and believed patients could become excited about AI-diagnostics with educational sessions (*D* = 0.15, *n* = 220, *p* < 0.001). Men were additionally more inclined to shift balance of slide interpretation towards AI-tools (*D* = 0.11, *n* = 219, *p* < 0.02), were more optimistic errors would become less frequent with implementation of a highly skilled independent AI tool (*D* = 0.19, *n* = 166, *p* < 0.001), and more likely to feel using AI tools would garner respect from colleagues (*D* = 0.15, *n* = 221, *p* < 0.001). Compared to community pathologists, academic pathologists were more optimistic about AI-tool integration (*D* = 0.14, *n* = 100, *p* < 0.001), more likely to adopt before formal validation/support attained (*D* = 0.25, *n* = 98, *p* < 0.001), and more likely to anticipate cost-effectiveness (*D* = 0.14, *n* = 101, *p* < 0.05), and quality assurance (*D* = 0.19, *n* = 83, *p* < 0.01). We did not find a significant difference between the distribution of male and female respondents in this later analysis to account for these differences (χ^2^ test, *p* = 0.33). When respondents were grouped into age over and under 40 years of age, respondents over 40 years old were more optimistic errors would become less frequent with implementation of a highly skilled independent (*D* = 0.12, *n* = 150, *p* < 0.05) or assistant AI tool (*D* = 0.13, *n* = 159, *p* < 0.01). Respondents over 40 predicted earlier integration of AI tools into their personal practice (*D* = 0.16, *n* = 196, *p* < 0.001), and pathology practice more broadly (*D* = 0.13, *n* = 197, *p* < 0.01). This result was somewhat unexpected and we believe it is confounded by the increased number of older respondents being male (66% (male) vs 49% (female) χ^2^ test, *P* < 0.001). Finally, given strong representation from British, American, and Canadian pathologists, we performed subgroup comparisons between these three countries. The only significant finding was that of slightly increased interest in AI technologies amongst US respondents compared to Canadian (*D* = 0.19, *n* = 114, *p* < 0.001), and somewhat reduced concern about potential job displacement from US respondents compared to Canadian (*D* = 0.16, *n* = 114, *p* < 0.01). Comparison of trainee responses (resident, fellow) to practicing respondents (staff pathologists, site directors, and department heads) failed to find significant differences. Similarly, subgroup comparisons between generalists (general pathology, anatomic pathology) and subspecialty pathologists (neuropathology, cardiovascular pathology, etc.) did not find significant associations or differences.

## Discussion

This survey was developed to begin gauging the pathology community’s perceptions, level of understanding, concerns, and opinions on the emerging use of AI in pathology practice, research, and training. To our knowledge, this is the largest published survey of the international pathology community of its kind. We do however acknowledge the shortcomings of the survey approach used in this study. In distributing this voluntary and anonymous survey by the methods described, it is not possible to calculate a survey response rate, or quantify non-response bias in our data. Certainly, the possibility that respondents are more likely than non-respondents to hold strong opinions or emotions on this issue is a real one, and should be considered. As a further limitation, respondents to our survey almost universally practice in North America and Europe, and so the perspectives of South American, African, and Australasian pathologists are significantly under-represented. Additionally, we appreciate that specific wording of some our survey questions have the potential to bias or polarize results to some degree. Several questions attempted to gauge respondents’ levels of excitement or concern, rather than dispassionately quantifying respondent’s predictions or perceptions of the likelihood of various scenarios (e.g., question 23: “Are you concerned that AI will eventually replace pathologists”). We used questions of this nature because we were interested in clearly identifying the emotional reactions and outlook of pathologists on AI, given extensive conversations in both media and academic literature about impacts of this technology on diagnostic specialties. However, we recognize that quantification of emotional reactions or outlook are not surrogate objective measures of pathologist’s predictions about implementation scenarios for AI in pathology. Our inability to definitively confirm the authenticity of respondents to our survey is an additional limitation (common to many large-scale surveys of this kind), and is acknowledged. Finally, while we did utilize KS analysis to evaluate differences in perspectives amongst our respondent demographic subgroups, we appreciate that these differences are likely to be fluid, and to change considerably as technologies evolve.

The common prediction of a relatively short interval to implementation of these tools suggests that early and pre-emptive measures to facilitate smooth implementation may be of significant value for forward-thinking pathology departments. For example, many respondents believe AI-tools will become useful for improvement of QA initiatives, suggesting a need to delineate how these technologies should be formally validated and adopted. Respondents frequently agreed that some education and training, either from an industry representative (41.1%) or through a dedicated course/workshop (29.0%) would help to facilitate safe and efficient adoption. Early efforts to implement educational presentations and formalized workshops may help to ease anxiety, increase awareness, and hopefully permit valuable pathologist-input into design and integration approaches. From a marketing perspective, these data suggest that platform developers will need to focus on demonstrating efficacy and safety of their technology, with little need to persuade pathologists that AI is a useful tool for their practices.

Several important concerns were raised by respondents in the comment sections (see Supplementary File 2). One important point raised by several respondents is that of limited utilization of digital pathology workflows in many institutions around the world. Digitized image data forms the basic informational input for AI-histology tools, and is a requisite for their use. Commonly cited barriers to integration of digital pathology include prohibitive cost analyses, perceived lack of impact on patient outcome, regulatory barriers, and reticence of pathologists due to concerns surrounding comfort, speed, and efficacy of digital systems.^27^ Even for institutions with digital pathology platforms, technical limitations such as lack of 3D whole slide scanners (many rely on 2D planar scanners), image variability between scanning devices, and technical artefacts may present additional hurdles.^28^ Interestingly, if AI diagnostic platforms can be shown to robustly improve diagnostic accuracy and efficiency for patients and hospitals, the elimination of these technical barriers (ie. implementation of a digital pathology platform) may become an expectation for any hospital committed to delivering world-class health care to its patients.

The legal implications of AI platforms in medicine, both from a regulatory and malpractice standpoint were a frequent theme amongst respondents. Considerable regulatory legislation can be expected to arise if a non-experimental AI-histology tool were brought to market, which may have implications for tool development and implementation. Additional concerns about attribution of medico-legal responsibility for errors occurring in AI-assisted workflows were raised, with some respondents concerned that platform vendors would not be held appropriately responsible for errors caused by their technology. Historically, a significant number of pathology errors precipitating malpractice suits have been those made in the analytic phase (i.e., false positive and false negative diagnoses), which conceivably could be both intercepted, and caused by AI-tool contributions.^29^ Regarding this issue, nearly half of respondents believed that pathology errors made in cases with AI-platform contributions, both pathologist and vendor should be held equally liable and a small minority even believed that the vendor alone should be responsible. The remaining half felt that responsibility should remain solely with the pathologist. Approaches to both regulatory and medicolegal aspects of AI-tool implementation remain unclear. Perhaps the most instructive legal precedents for these issues will be found in the burgeoning field of autonomous vehicles, where lawmakers, industry representatives, and the public are working through the legal implications of self-driving technologies.^30^

Examining our data in retrospect, there are a number of important questions which should be considered by future investigators performing surveys of a similar nature. Understanding the perspectives of pathologists on how reimbursement schemas should adapt to the implementation of AI-based tools is of considerable importance to all stakeholders in the field. Designing questions to achieve insight into which individuals or demographic groups would be most likely to be early adaptors of these tools could focus attention on these groups, and ensure that ‘early experiences’ are captured and evaluated for broader benefit of the community. Surveying pathologists more specifically on how AI-tools could be integrated into their personal clinical practice would highlight areas of focus for developers and hospitals. Finally, the evolution of the relationship between histopathology and AI tools may be informed heavily by the availability of pathologists in a particular region or institution. For example, a 2017–2018 workforce census in the UK has identified staff shortages as a critical concern,^31^ and thoroughly investigating perspectives of providers in these practice settings on how AI tools relate to these challenges would be valuable. Of note, subgroup differences were not identified between UK pathologists and their North American counterparts in our survey data.

In conclusion, we present a survey of pathology colleagues around the world from a variety of demographic and practice backgrounds. While we identified some differences in perspectives depending on age, gender, and practice type, there appears to be general enthusiasm amongst respondents for the use of AI as a diagnostic tool in pathology practice, and concern about impacts on job security is relatively low. Most respondents envision the eventual implementation of AI-tools as decision support tools used by human diagnosticians, not in place of. Several concerns about technological barriers, especially limited current day usage of digital pathology platforms in many centres, and concerns about AI tool errors and medico-legal responsibility were raised, suggesting that these will require further attention before effective implementation can be achieved.

## Methods

### Analytical methods

An anonymized survey consisting of 43 questions was created using the online SurveyMonkey tool (https://www.surveymonkey.com) (Supplementary File 1). Pathologists listed as faculty members on Canadian university websites were invited by email to complete the survey. Given limited response using this approach, we created a generic link and requested it be shared amongst pathologists at pathology departments throughout Canada. We additionally shared the survey on the Twitter platform, at meeting presentations, and on the popular neuropathology blog (http://neuropathologyblog.blogspot.com/). Further, we contacted 95 pathology associations worldwide to request distribution of our survey to their membership, of which the Association for Molecular Pathology (AMP), Association for Pathology Informatics (API), and the European Society of Pathology (ESP) agreed to distribute the survey. These efforts to achieve maximal distribution resulted in a final survey respondent count of 487 individuals. All responses were obtained voluntarily (i.e., not institutionally enforced) from professional colleagues and anonymized (de-identified, aggregated, and tabulated) prior to analysis. This study, therefore, met the exclusion criteria of the Canadian Tri-Council Policy Statement for research necessitating a review by an institutional research ethics board. Survey results were tallied for each question and described as an aggregate percentage result for reporting and discussion. Two sample Kolmogorov–Smirnov testing was performed to estimate statistically significant associations between demographic parameters (age, gender, practice type, and career stage) and patterns of responses.^32^

## Supplementary information

Supplementary Information File

## Data Availability

Anonymized aggregate data are provided in the supplementary files; individual survey responses have not been made publicly available to maintain respondent privacy and confidentiality

## Supplementary information

**Supplementary information** accompanies the paper on the *npj Digital Medicine* website (10.1038/s41746-019-0106-0).

## Code availability

All analysis was performed on commercially available software tools (SurveyMonkey, Microsoft Excel).
