# Supplementary material for: Physician perspectives on integration of artificial intelligence into diagnostic pathology
Source: NPJ Digit Med. 2019 Apr 26;2:28. doi: 10.1038/s41746-019-0106-0 (PMC6550202; doi:10.1038/s41746-019-0106-0)
Supplement: Supplementary file 1 — Supplementary Information File [file 41746_2019_106_MOESM1_ESM.pdf]

Supplementary File 1 – Summary of all Survey Responses

Q1 Have you completed this survey before?

Answered: 475 Skipped: 5

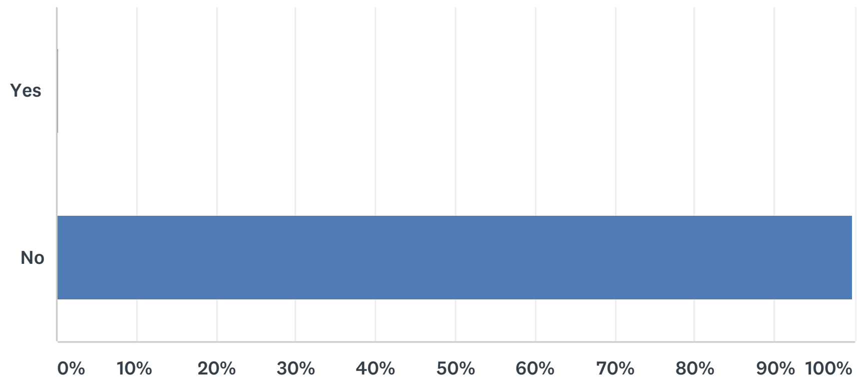

ANSWER CHOICES	RESPONSES	
Yes	0.21%	1
No	99.79%	474
TOTAL		475

Q2 What is your age?

Answered: 477 Skipped: 3

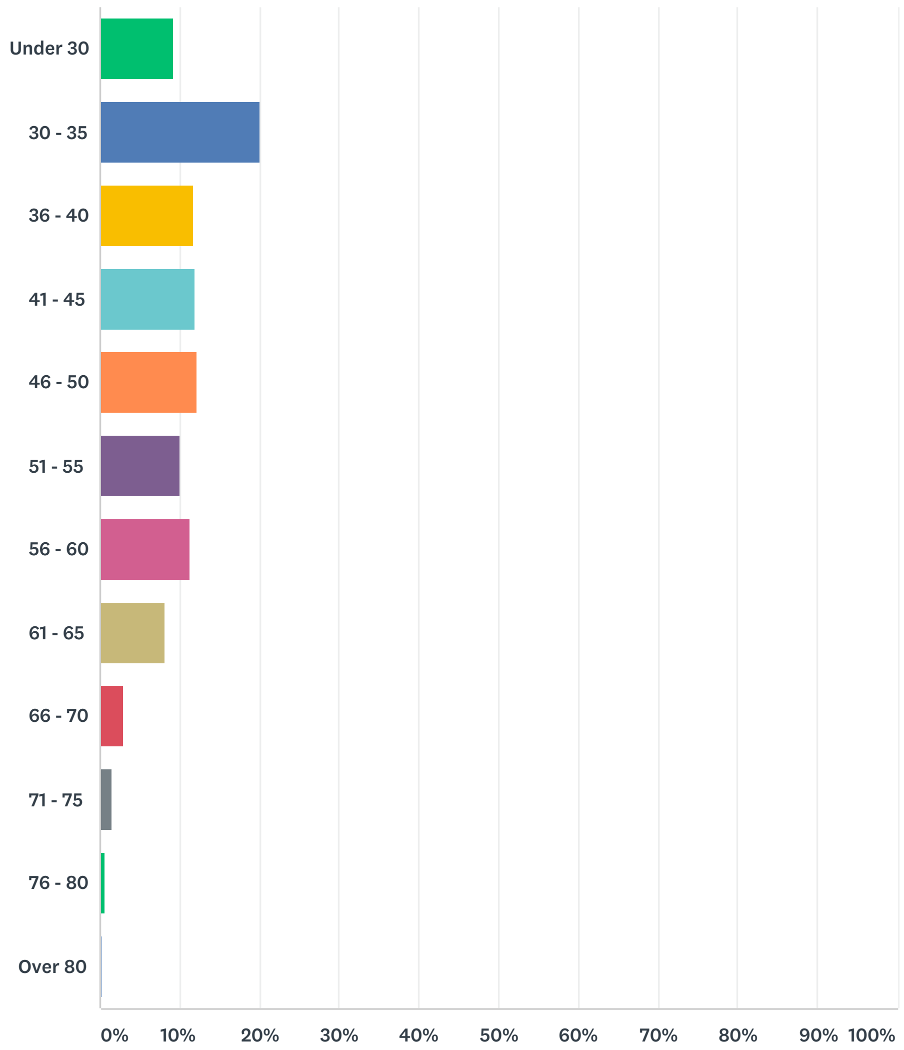

ANSWER CHOICES	RESPONSES	
Under 30	9.22%	44
30 - 35	20.13%	96
36 - 40	11.74%	56
41 - 45	11.95%	57
46 - 50	12.16%	58
51 - 55	10.06%	48
56 - 60	11.32%	54
61 - 65	8.18%	39
66 - 70	2.94%	14
71 - 75	1.47%	7
76 - 80	0.63%	3

Over 80	0.21%	1
TOTAL		477

Q3 What is your sex?

Answered: 477 Skipped: 3

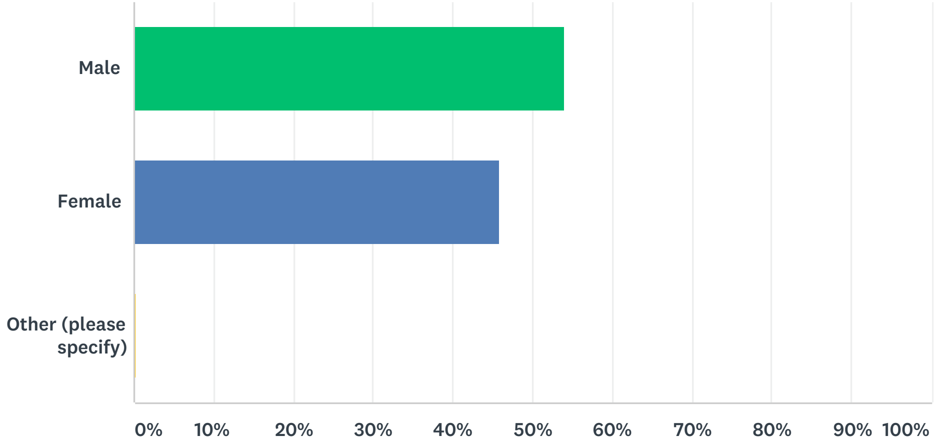

ANSWER CHOICES	RESPONSES	
Male	53.88%	257
Female	45.91%	219
Other (please specify)	0.21%	1
TOTAL		477

Q4 Which best describes your level of training?

Answered: 479 Skipped: 1

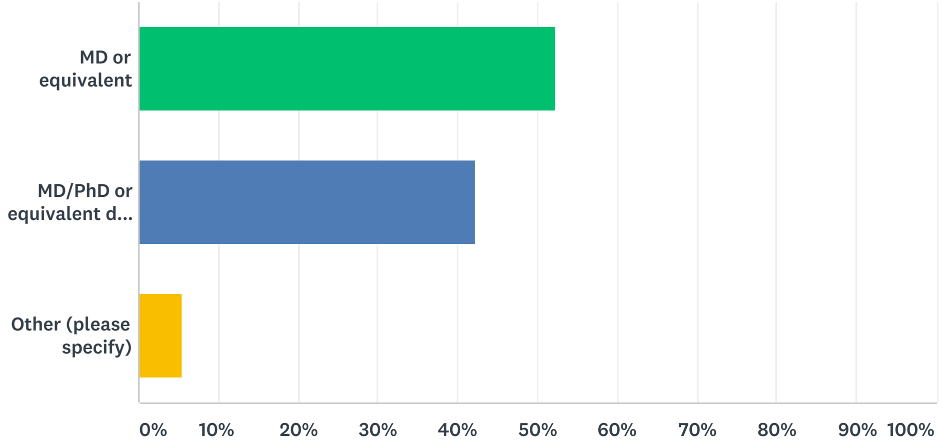

ANSWER CHOICES	RESPONSES	
MD or equivalent	52.40%	251
MD/PhD or equivalent dual degree	42.17%	202
Other (please specify)	5.43%	26
TOTAL		479

Q5 Which best describes your current position?

Answered: 479 Skipped: 1

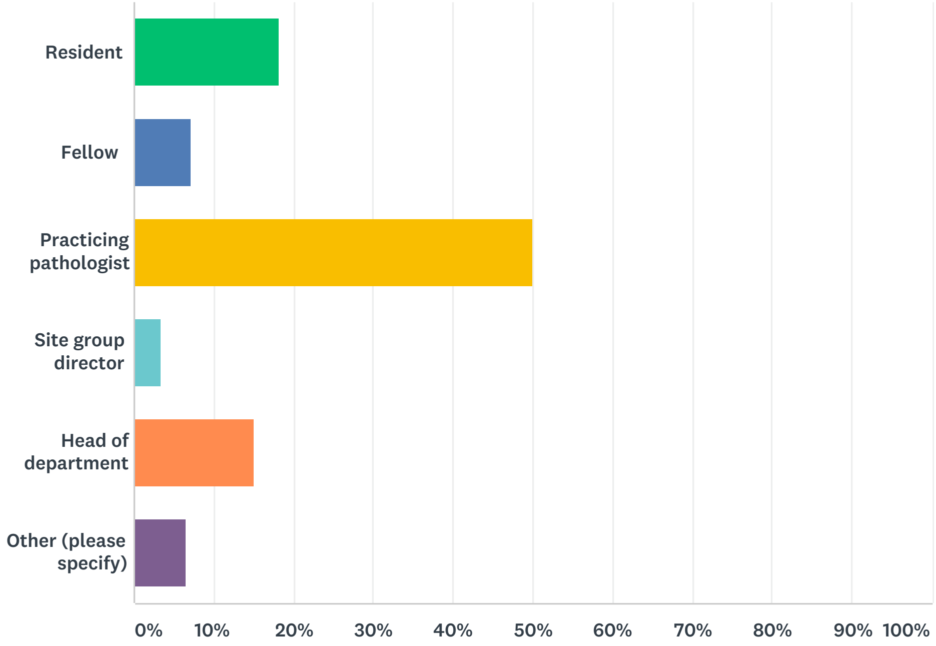

ANSWER CHOICES	RESPONSES	
Resident	18.16%	87
Fellow	7.10%	34
Practicing pathologist	49.90%	239
Site group director	3.34%	16
Head of department	15.03%	72
Other (please specify)	6.47%	31
TOTAL		479

Q6 What subspecialty of pathology do you practice or are training in?

Answered: 476 Skipped: 4

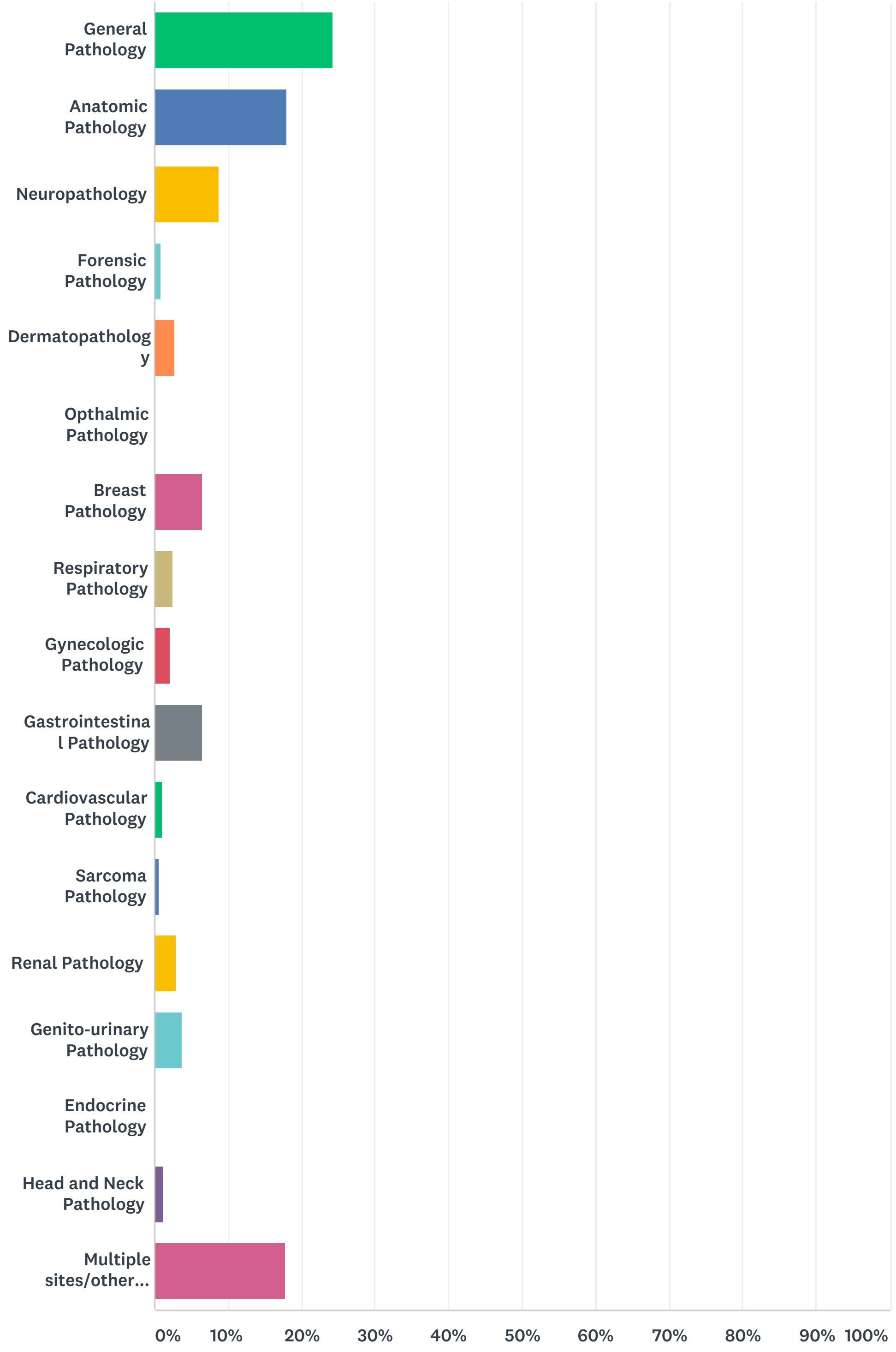

ANSWER CHOICES	RESPONSES	
General Pathology	24.37%	116
Anatomic Pathology	18.07%	86
Neuropathology	8.82%	42

Forensic Pathology	0.84%	4
Dermatopathology	2.73%	13
Opthalmic Pathology	0.00%	0
Breast Pathology	6.51%	31
Respiratory Pathology	2.52%	12
Gynecologic Pathology	2.10%	10
Gastrointestinal Pathology	6.51%	31
Cardiovascular Pathology	1.05%	5
Sarcoma Pathology	0.63%	3
Renal Pathology	2.94%	14
Genito-urinary Pathology	3.78%	18
Endocrine Pathology	0.00%	0
Head and Neck Pathology	1.26%	6
Multiple sites/other (please specify)	17.86%	85
TOTAL		476

Q7 In what type of setting do you currently practice?

Answered: 478 Skipped: 2

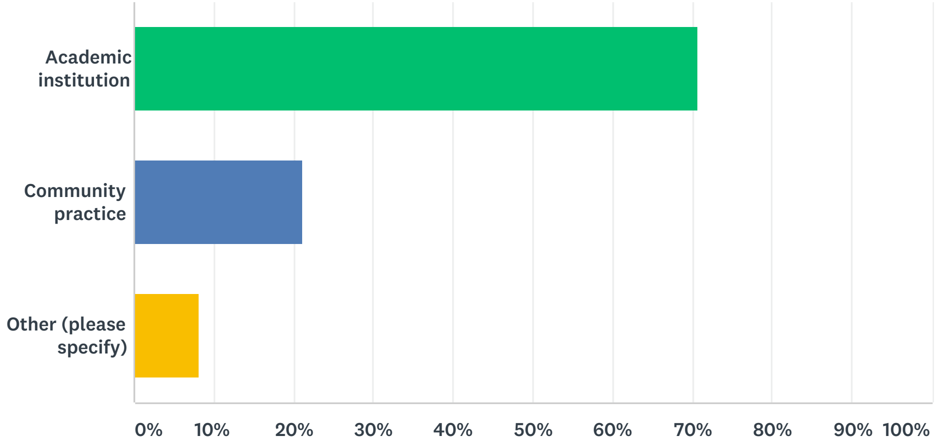

ANSWER CHOICES	RESPONSES	
Academic institution	70.71%	338
Community practice	21.13%	101
Other (please specify)	8.16%	39
TOTAL		478

Q8 How large is the department of your primary appointment?

Answered: 478 Skipped: 2

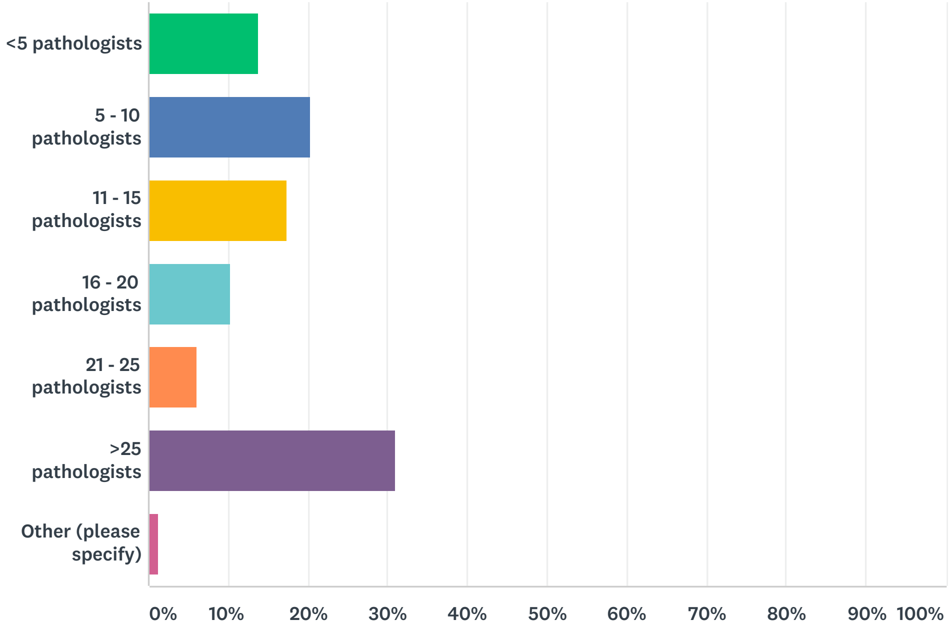

ANSWER CHOICES	RESPONSES	
<5 pathologists	13.81%	66
5 - 10 pathologists	20.29%	97
11 - 15 pathologists	17.36%	83
16 - 20 pathologists	10.25%	49
21 - 25 pathologists	6.07%	29
>25 pathologists	30.96%	148
Other (please specify)	1.26%	6
TOTAL		478

Q9 What country are you currently practicing/training in?

Answered: 475 Skipped: 5

Q10 What city are you currently practicing/training in?

Answered: 466 Skipped: 14

Q11 How long have you been practicing pathology? (Include years in residency training)

Answered: 474 Skipped: 6

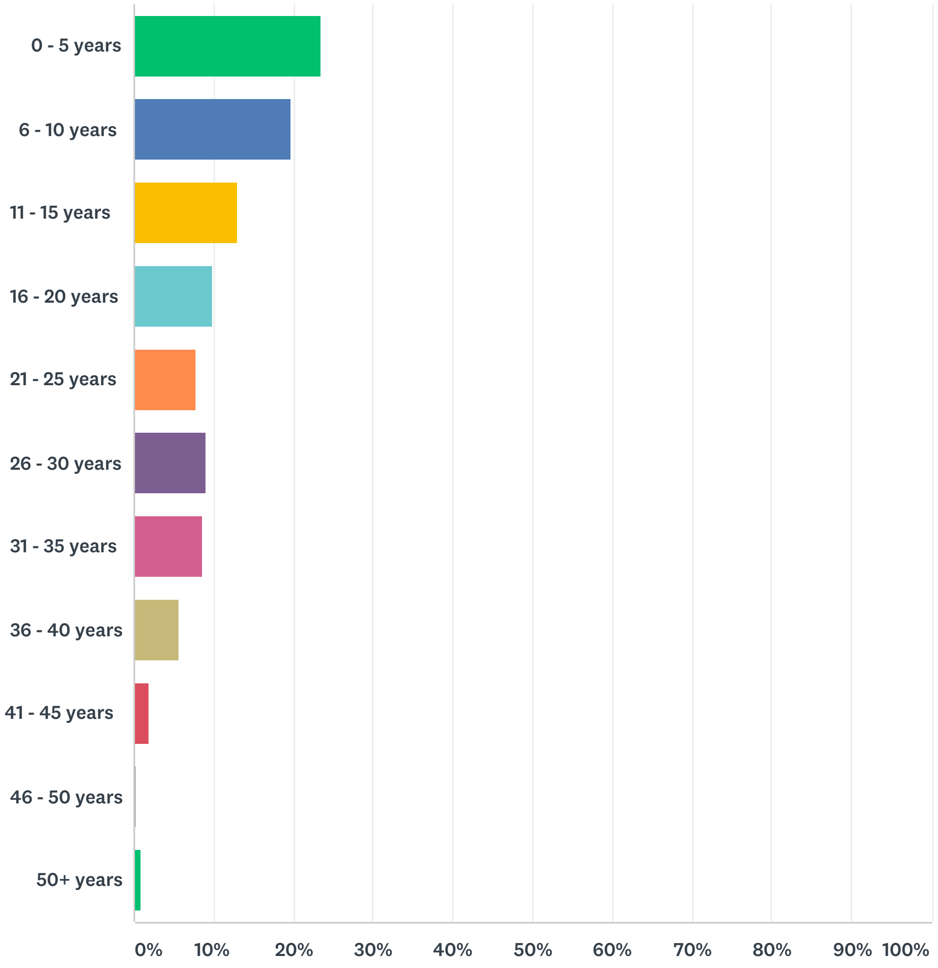

ANSWER CHOICES	RESPONSES	
0 - 5 years	23.42%	111
6 - 10 years	19.62%	93
11 - 15 years	12.87%	61
16 - 20 years	9.92%	47
21 - 25 years	7.81%	37
26 - 30 years	9.07%	43
31 - 35 years	8.65%	41
36 - 40 years	5.70%	27
41 - 45 years	1.90%	9
46 - 50 years	0.21%	1
50+ years	0.84%	4
TOTAL		474

Q12 What percentage of your time is devoted to clinical practice? (Note: the sum of questions 12 - 15 should add up to 100%)

Answered: 477 Skipped: 3

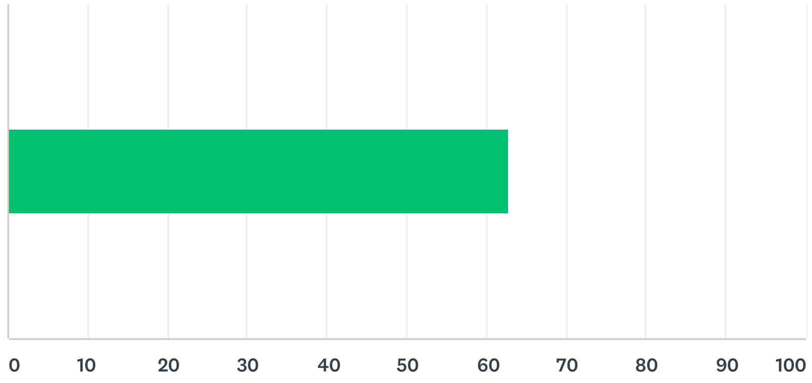

ANSWER CHOICES	AVERAGE NUMBER	TOTAL NUMBER	RESPONSES
	63	29,944	477
Total Respondents: 477			

Q13 What percentage of your time is devoted to teaching? (Note: the sum of questions 12 - 15 should add up to 100%)

Answered: 465 Skipped: 15

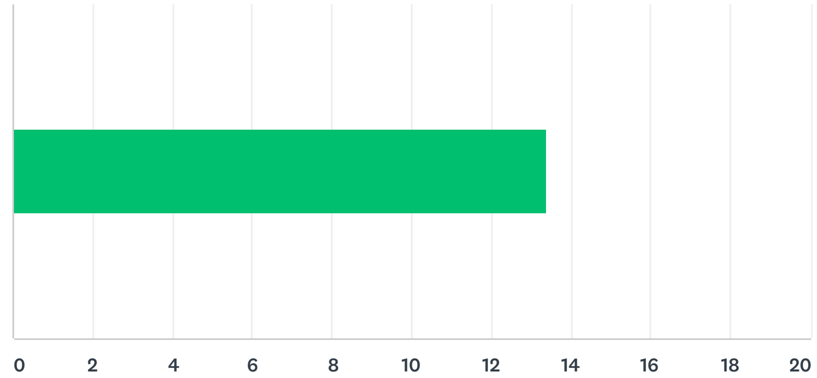

ANSWER CHOICES	AVERAGE NUMBER	TOTAL NUMBER	RESPONSES
	13	6,227	465
Total Respondents: 465			

Q14 What percentage of your time is devoted to administrative activities? (Note: the sum of questions 12 - 15 should add up to 100%)

Answered: 459 Skipped: 21

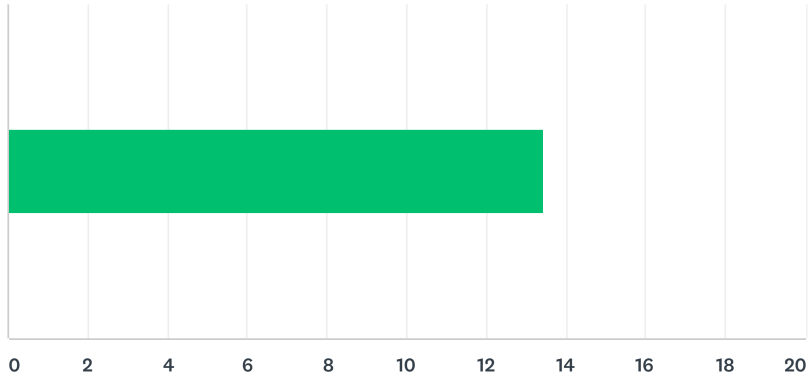

ANSWER CHOICES	AVERAGE NUMBER	TOTAL NUMBER	RESPONSES
	13	6,158	459
Total Respondents: 459			

Q15 What percentage of your time is devoted to research activities? (Note: the sum of questions 12 - 15 should add up to 100%)

Answered: 456 Skipped: 24

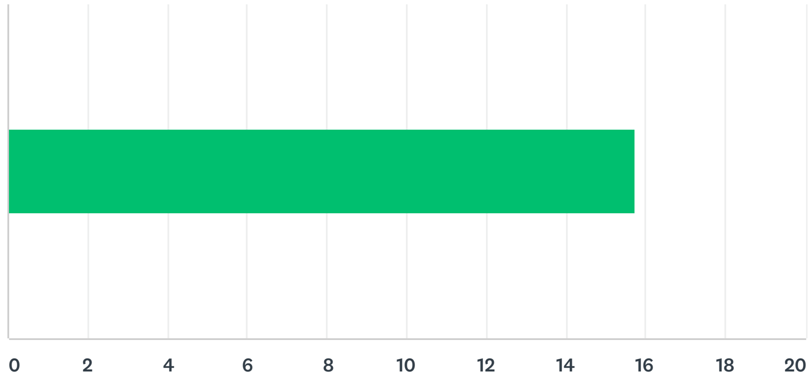

ANSWER CHOICES	AVERAGE NUMBER	TOTAL NUMBER	RESPONSES
	16	7,177	456
Total Respondents: 456			

Q16 How do you currently use digital images in your practice/training? (Check all that apply)

Answered: 475 Skipped: 5

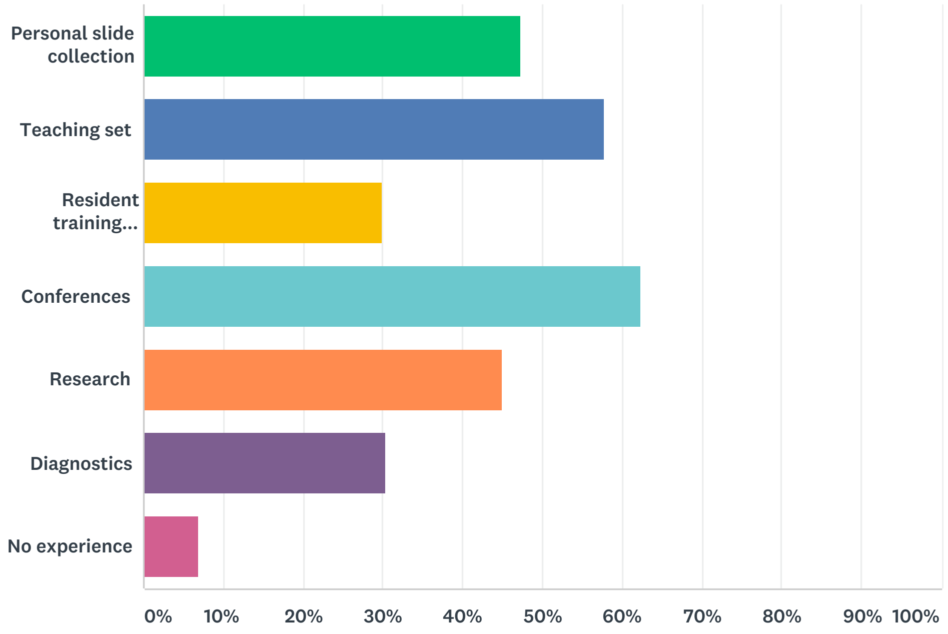

ANSWER CHOICES	RESPONSES	
Personal slide collection	47.37%	225
Teaching set	57.68%	274
Resident training examinations	29.89%	142
Conferences	62.32%	296
Research	45.05%	214
Diagnostics	30.32%	144
No experience	6.95%	33
Total Respondents: 475		

Q17 Approximately how many talks/papers have you attended/read that focus on using computer image analysis to make interpretations of diagnostic or prognostic patterns? (e.g. find micro-metastases, estimate proliferation index, etc.)

Answered: 473 Skipped: 7

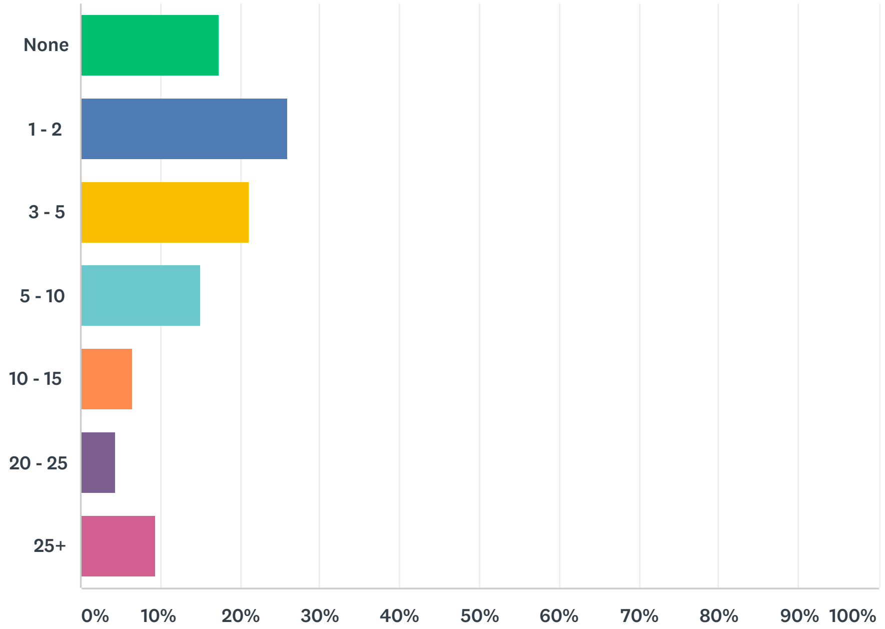

ANSWER CHOICES	RESPONSES	
None	17.34%	82
1 - 2	26.00%	123
3 - 5	21.14%	100
5 - 10	15.01%	71
10 - 15	6.55%	31
20 - 25	4.44%	21
25+	9.51%	45
TOTAL		473

Q18 How do you describe your use of genomic/molecular diagnostic tests (e.g. methylation, FISH, etc.) in your practice compared to other pathologists you work with?

Answered: 472 Skipped: 8

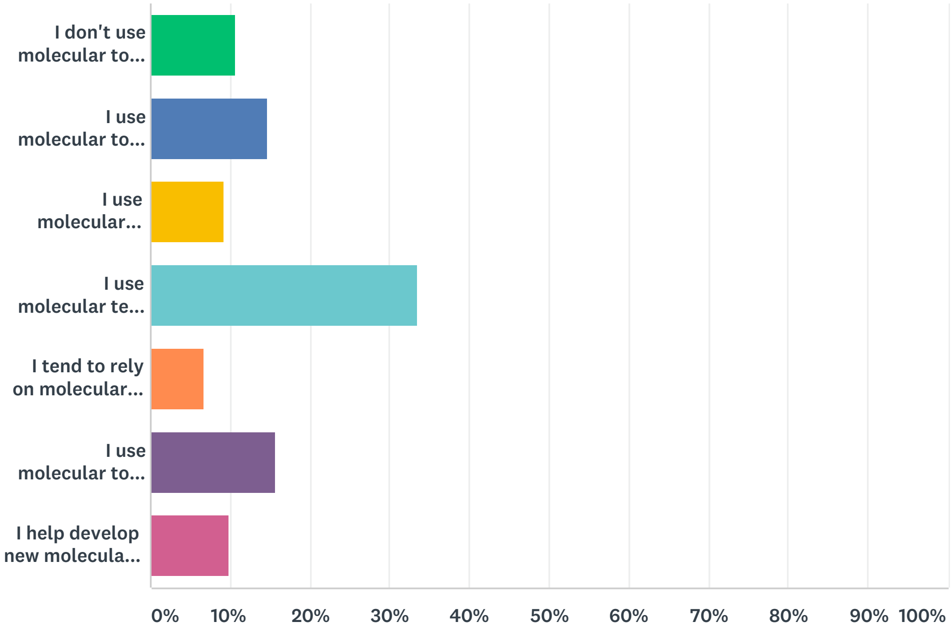

ANSWER CHOICES	RESPONSES	
I don't use molecular tools (budgetary constraints)	10.59%	50
I use molecular tools only when absolutely necessary	14.62%	69
I use molecular tools, but to a lesser extent than my colleagues	9.11%	43
I use molecular tests about the same as my colleagues	33.47%	158
I tend to rely on molecular tools more than my colleagues	6.78%	32
I use molecular tools on almost all appropriate cases	15.68%	74
I help develop new molecular tools for research and clinical use	9.75%	46
TOTAL		472

Q19 What is your understanding of the role of AI in pathological analysis? (Check all that apply)

Answered: 477 Skipped: 3

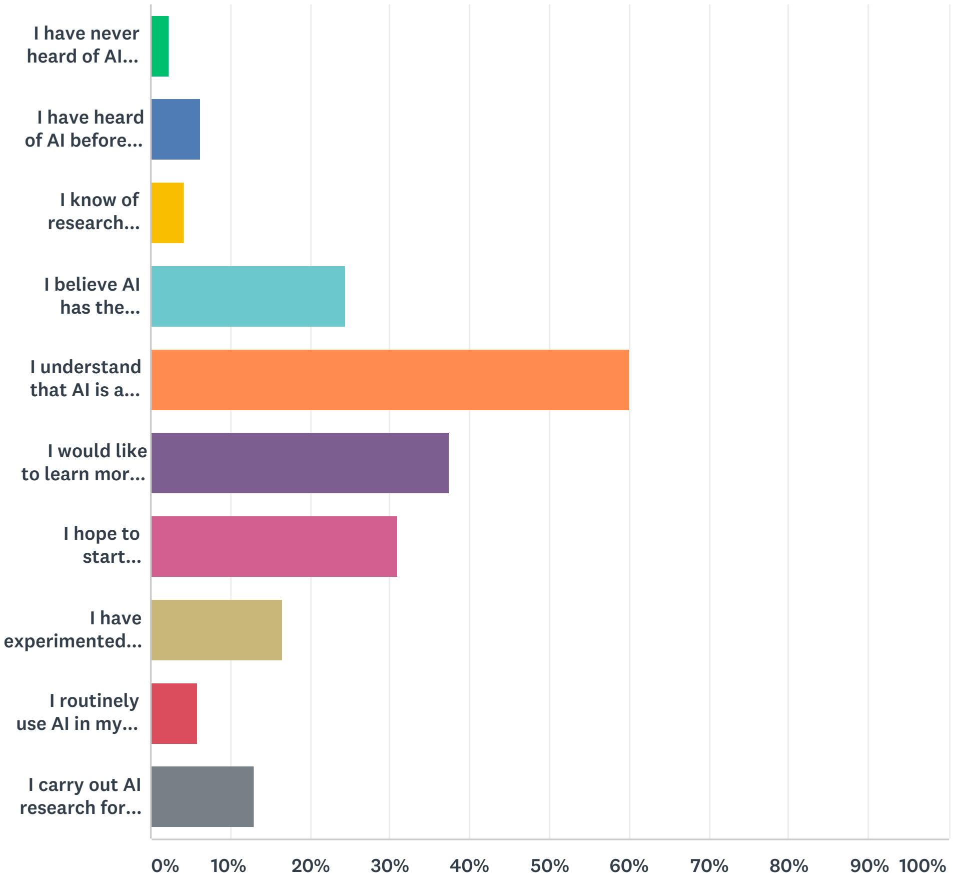

ANSWER CHOICES	RESPONSES	
I have never heard of AI before	2.31%	11
I have heard of AI before but didn't realize it had applications to pathology	6.29%	30
I know of research applications of AI in pathology but I don't think it will reach clinical practice	4.19%	20
I believe AI has the potential to eventually become a tool in pathology, but not anytime soon	24.53%	117
I understand that AI is a new and emerging tool in pathology	59.96%	286
I would like to learn more about how I can start experimenting with AI in pathology	37.53%	179
I hope to start experimenting with programs that use AI to carry out specific tasks in pathology	31.03%	148
I have experimented with programs that use AI to carry out specific tasks in pathology	16.56%	79
I routinely use AI in my clinical practice to carry out specific tasks (e.g. Ki67 estimates)	5.87%	28
I carry out AI research for pathology application	13.00%	62
Total Respondents: 477		

Q20 In general, how do you describe your willingness to incorporate new technological advances into your practice?

Answered: 475 Skipped: 5

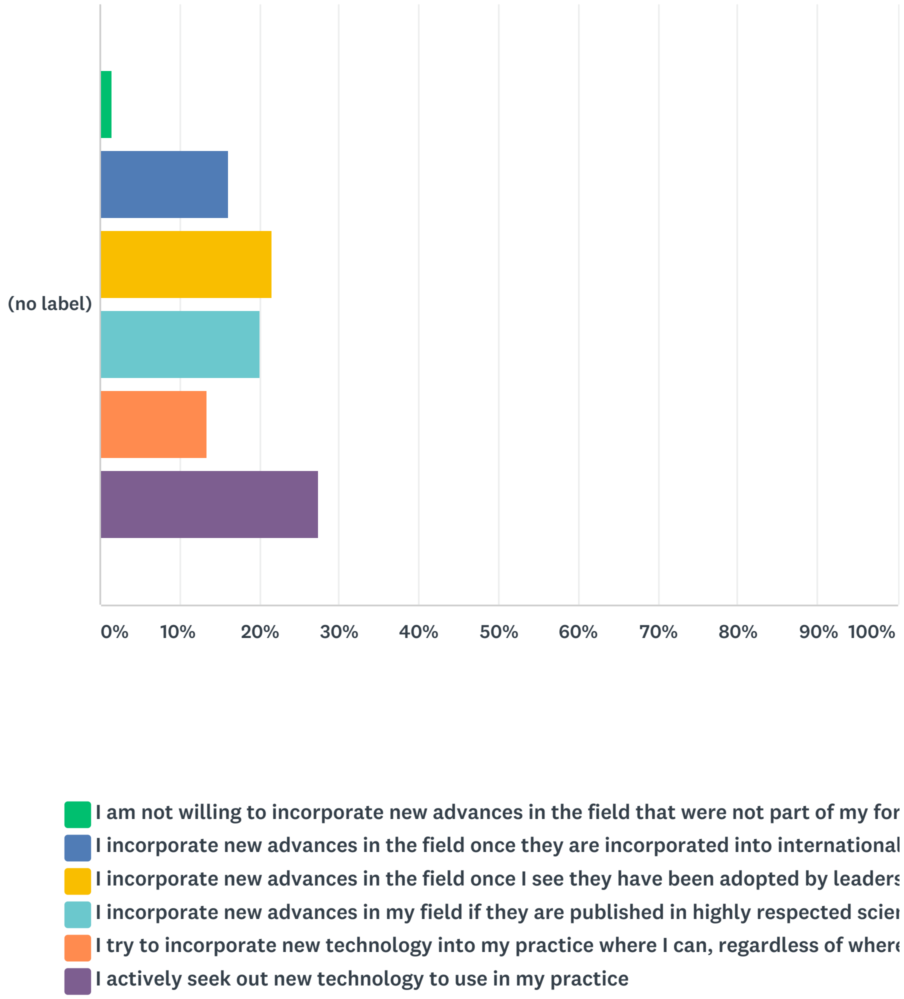

	I AM NOT WILLING TO INCORPORATE NEW ADVANCES IN THE FIELD THAT WERE NOT PART OF MY FORMAL TRAINING	I INCORPORATE NEW ADVANCES IN THE FIELD ONCE THEY ARE INCORPORATED INTO INTERNATIONAL GUIDELINES	I INCORPORATE NEW ADVANCES IN THE FIELD ONCE I SEE THEY HAVE BEEN ADOPTED BY LEADERS IN MY FIELD	I INCORPORATE NEW ADVANCES IN MY FIELD IF THEY ARE PUBLISHED IN HIGHLY RESPECTED SCIENTIFIC JOURNALS	I TRY TO INCORPORATE NEW TECHNOLOGY INTO MY PRACTICE WHERE I CAN, REGARDLESS OF WHERE IT'S PUBLISHED	I ACTIVELY SEEK OUT NEW TECHNOLOGY TO USE IN MY PRACTICE	TOTAL	WEIGH AVERA
(no label)	1.47%<br>7	16.21%<br>77	21.47%<br>102	20.00%<br>95	13.47%<br>64	27.37%<br>130	475	

Q21 How do you feel about the incorporation of AI technology into pathological analysis?

Answered: 476 Skipped: 4

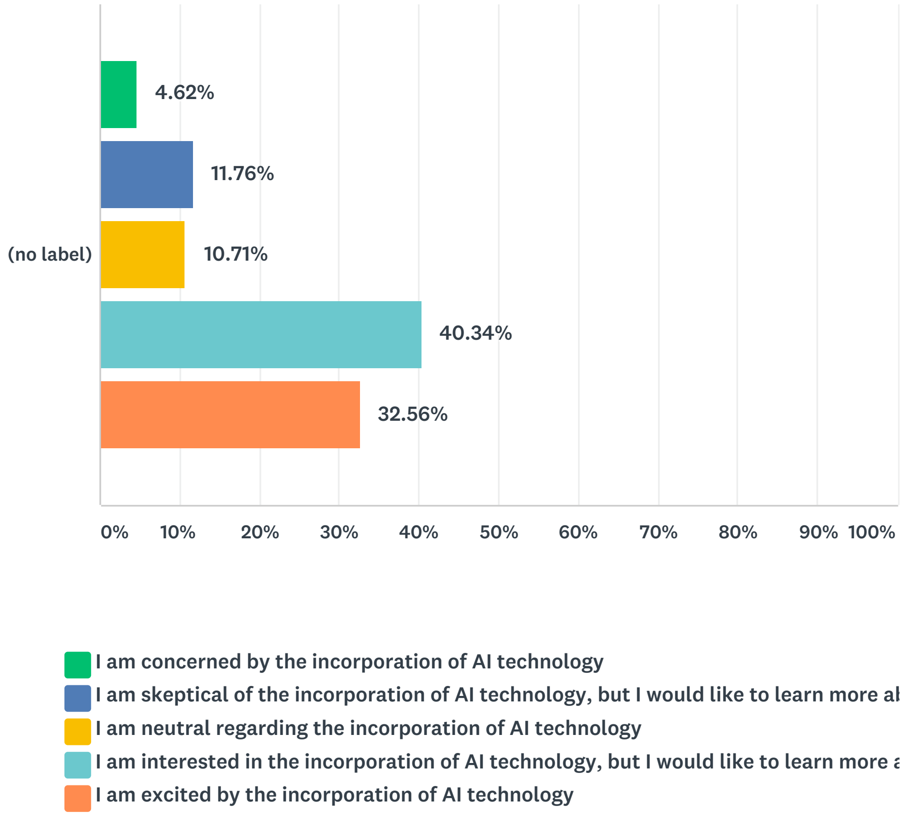

	I AM CONCERNED BY THE INCORPORATION OF AI TECHNOLOGY	I AM SKEPTICAL OF THE INCORPORATION OF AI TECHNOLOGY, BUT I WOULD LIKE TO LEARN MORE ABOUT IT	I AM NEUTRAL REGARDING THE INCORPORATION OF AI TECHNOLOGY	I AM INTERESTED IN THE INCORPORATION OF AI TECHNOLOGY, BUT I WOULD LIKE TO LEARN MORE ABOUT IT	I AM EXCITED BY THE INCORPORATION OF AI TECHNOLOGY	TOTAL	WEIGHTED AVERAGE
(no label)	4.62%<br>22	11.76%<br>56	10.71%<br>51	40.34%<br>192	32.56%<br>155	476	3.84

Q22 If an AI-based decision support tool could help improve your efficiency to the point where you could devote more time to other academic and/or research activities, would you want it available to you?

Answered: 473 Skipped: 7

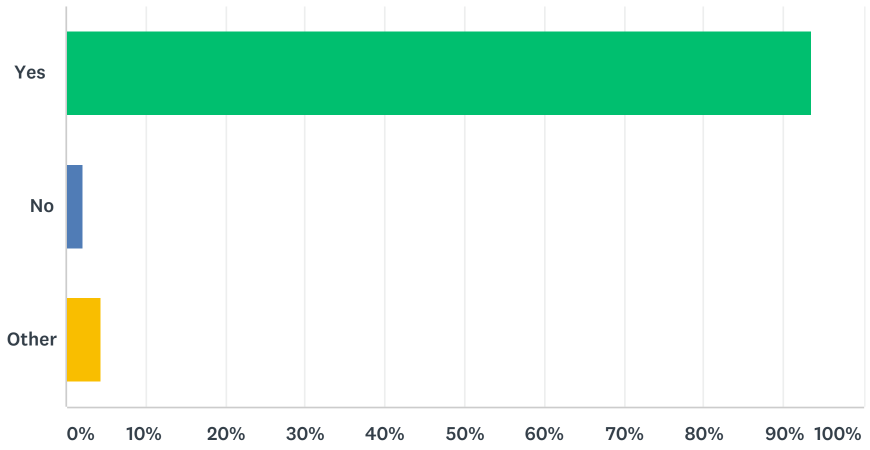

ANSWER CHOICES	RESPONSES	
Yes	93.45%	442
No	2.11%	10
Other	4.44%	21
TOTAL		473

Q23 Are you concerned that AI will eventually replace pathologists?

Answered: 477 Skipped: 3

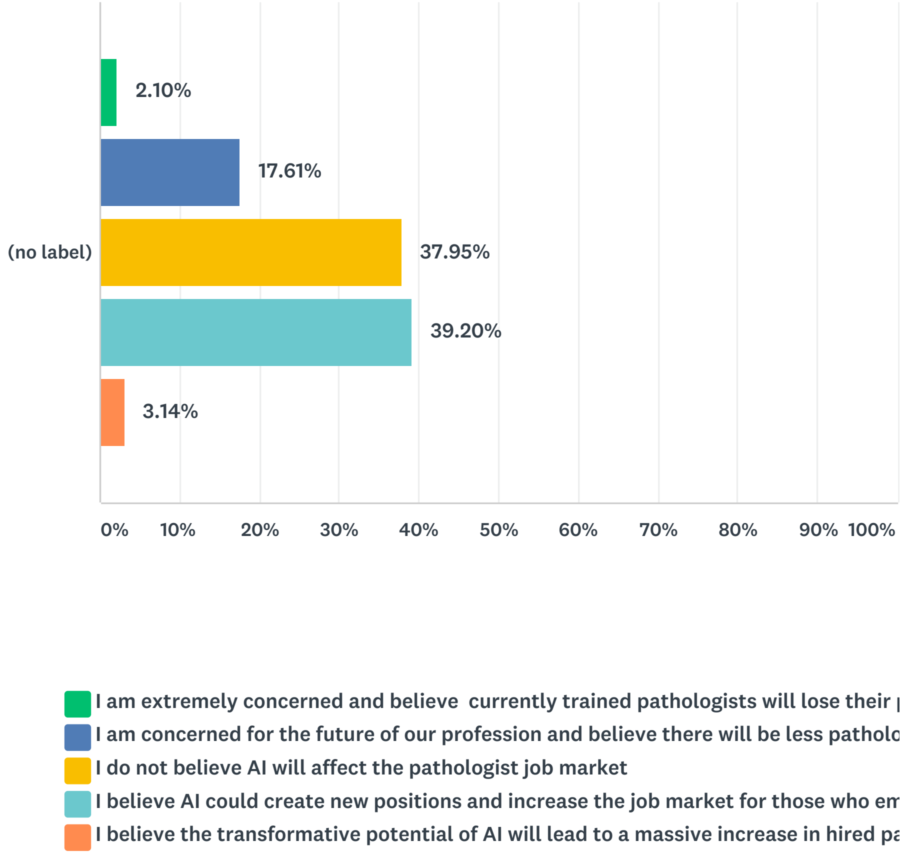

	I AM EXTREMELY CONCERNED AND BELIEVE CURRENTLY TRAINED PATHOLOGISTS WILL LOSE THEIR POSITIONS	I AM CONCERNED FOR THE FUTURE OF OUR PROFESSION AND BELIEVE THERE WILL BE LESS PATHOLOGISTS TRAINED	I DO NOT BELIEVE AI WILL AFFECT THE PATHOLOGIST JOB MARKET	I BELIEVE AI COULD CREATE NEW POSITIONS AND INCREASE THE JOB MARKET FOR THOSE WHO EMBRACE IT	I BELIEVE THE TRANSFORMATIVE POTENTIAL OF AI WILL LEAD TO A MASSIVE INCREASE IN HIRED PATHOLOGISTS	TOTAL	WEIGHTED AVERAGE
(no label)	2.10%<br>10	17.61%<br>84	37.95%<br>181	39.20%<br>187	3.14%<br>15	477	3.24

Q24 In the near future (5 years), do you believe the use of AI in pathological analysis will bring down the cost of diagnostic pathology?

Answered: 474 Skipped: 6

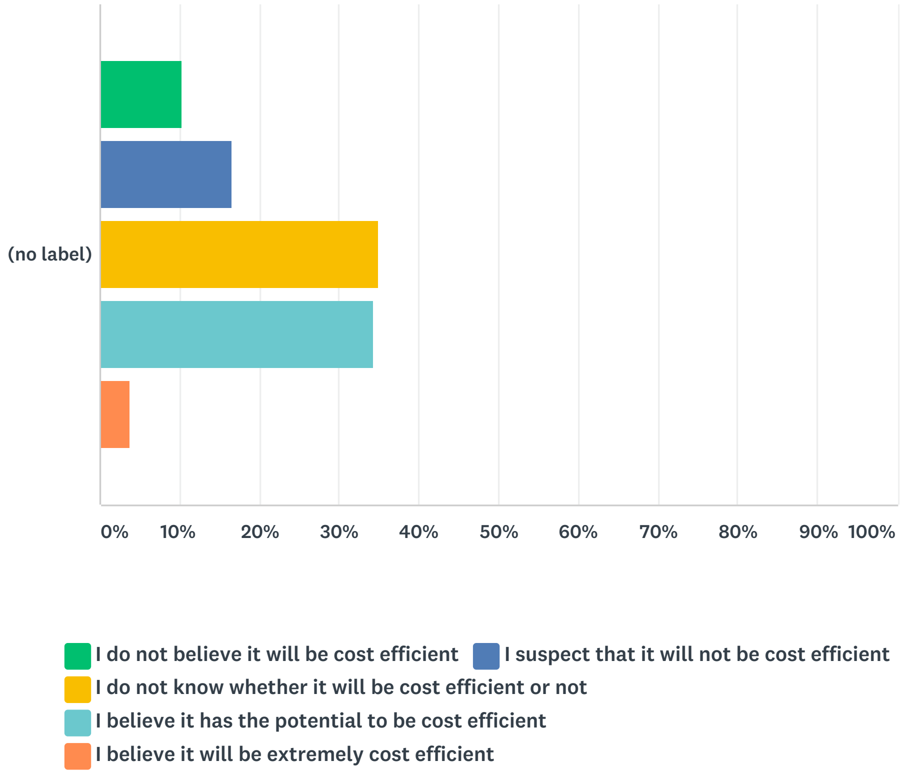

	I DO NOT BELIEVE IT WILL BE COST EFFICIENT	I SUSPECT THAT IT WILL NOT BE COST EFFICIENT	I DO NOT KNOW WHETHER IT WILL BE COST EFFICIENT OR NOT	I BELIEVE IT HAS THE POTENTIAL TO BE COST EFFICIENT	I BELIEVE IT WILL BE EXTREMELY COST EFFICIENT	TOTAL	WEIGHTED AVERAGE
(no label)	10.34%<br>49	16.46%<br>78	35.02%<br>166	34.39%<br>163	3.80%<br>18	474	3.05

Q25 How do you think integration of AI in pathology will affect your efficiency?

Answered: 476 Skipped: 4

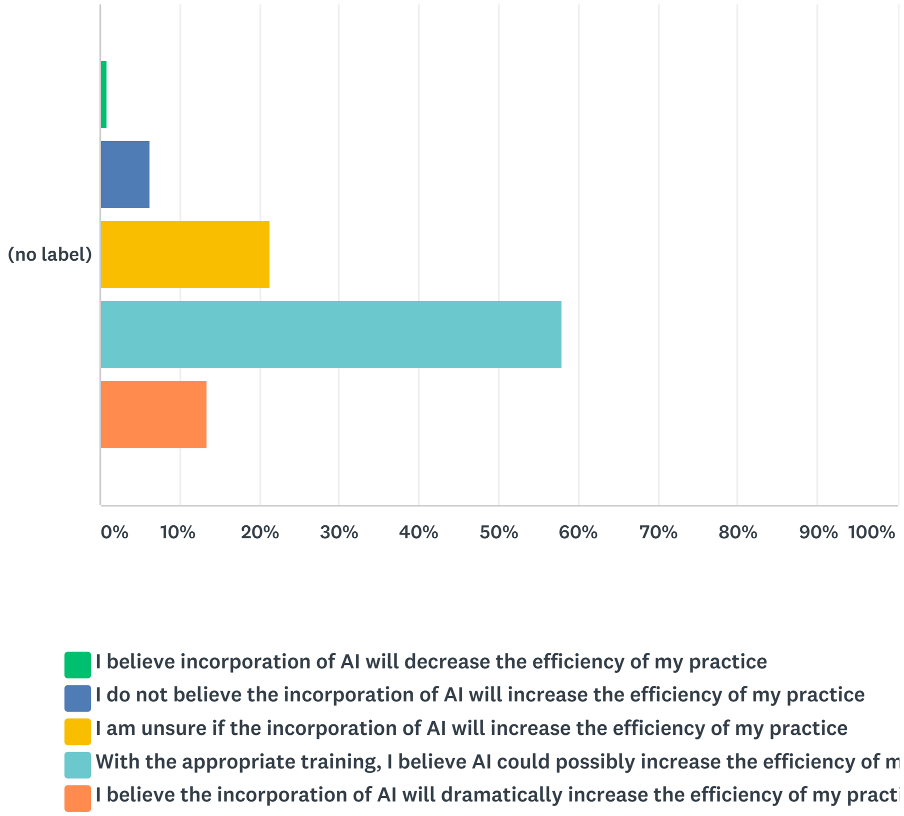

	I BELIEVE INCORPORATION OF AI WILL DECREASE THE EFFICIENCY OF MY PRACTICE	I DO NOT BELIEVE THE INCORPORATION OF AI WILL INCREASE THE EFFICIENCY OF MY PRACTICE	I AM UNSURE IF THE INCORPORATION OF AI WILL INCREASE THE EFFICIENCY OF MY PRACTICE	WITH THE APPROPRIATE TRAINING, I BELIEVE AI COULD POSSIBLY INCREASE THE EFFICIENCY OF MY PRACTICE	I BELIEVE THE INCORPORATION OF AI WILL DRAMATICALLY INCREASE THE EFFICIENCY OF MY PRACTICE	TOTAL	WEIGHTED AVERAGE
(no label)	0.84%<br>4	6.30%<br>30	21.43%<br>102	57.98%<br>276	13.45%<br>64	476	3.77

Q26 If AI is proven to be a valuable and safe decision support tool for pathologists, will you feel comfortable (from a training perspective) using AI tools in your practice?

Answered: 473 Skipped: 7

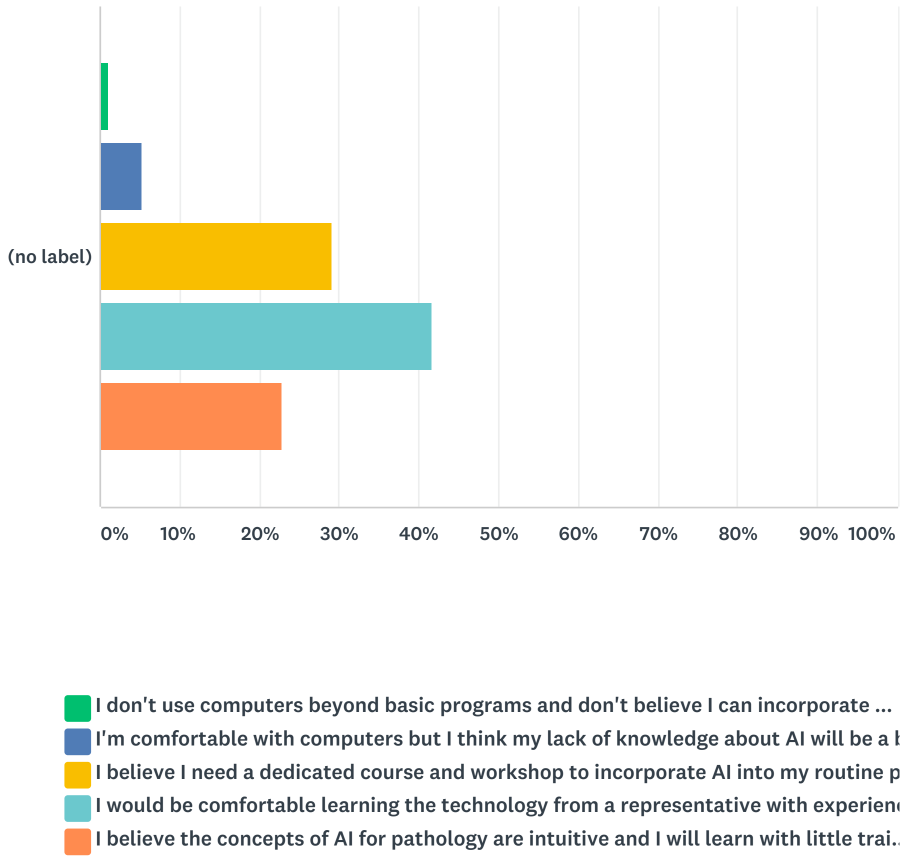

	I DON'T USE COMPUTERS BEYOND BASIC PROGRAMS AND DON'T BELIEVE I CAN INCORPORATE AI INTO MY PRACTICE	I'M COMFORTABLE WITH COMPUTERS BUT I THINK MY LACK OF KNOWLEDGE ABOUT AI WILL BE A BARRIER	I BELIEVE I NEED A DEDICATED COURSE AND WORKSHOP TO INCORPORATE AI INTO MY ROUTINE PRACTICE	I WOULD BE COMFORTABLE LEARNING THE TECHNOLOGY FROM A REPRESENTATIVE WITH EXPERIENCE IN THAT AREA	I BELIEVE THE CONCEPTS OF AI FOR PATHOLOGY ARE INTUITIVE AND I WILL LEARN WITH LITTLE TRAINING	TOTAL	WEIGHTED AVERAGE
(no label)	1.06%<br>5	5.29%<br>25	29.18%<br>138	41.65%<br>197	22.83%<br>108	473	3.80

Q27 How do you think the use of AI in your practice will be received by your clinical colleagues?

Answered: 474 Skipped: 6

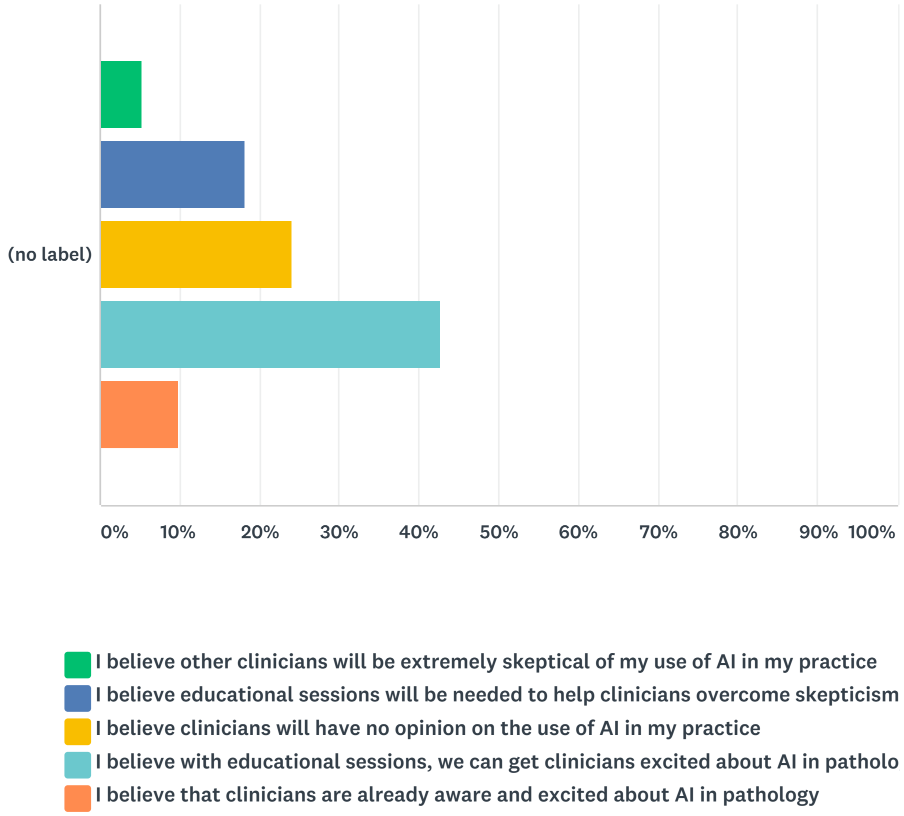

	I BELIEVE OTHER CLINICIANS WILL BE EXTREMELY SKEPTICAL OF MY USE OF AI IN MY PRACTICE	I BELIEVE EDUCATIONAL SESSIONS WILL BE NEEDED TO HELP CLINICIANS OVERCOME SKEPTICISM	I BELIEVE CLINICIANS WILL HAVE NO OPINION ON THE USE OF AI IN MY PRACTICE	I BELIEVE WITH EDUCATIONAL SESSIONS, WE CAN GET CLINICIANS EXCITED ABOUT AI IN PATHOLOGY	I BELIEVE THAT CLINICIANS ARE ALREADY AWARE AND EXCITED ABOUT AI IN PATHOLOGY	TOTAL	WEIGHTED AVERAGE
(no label)	5.27%<br>25	18.14%<br>86	24.05%<br>114	42.62%<br>202	9.92%<br>47	474	3.34

Q28 How do you think the use of AI in your practice will be received by your patients?

Answered: 475 Skipped: 5

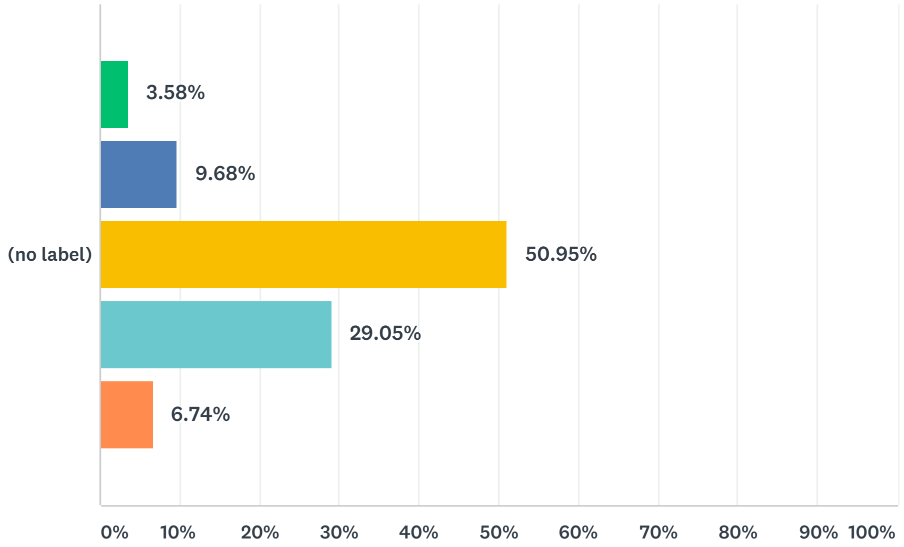

	I BELIEVE PATIENTS WILL BE EXTREMELY SKEPTICAL OF MY USE OF AI IN MY PRACTICE	I BELIEVE EDUCATIONAL SESSIONS WILL BE NEEDED TO HELP PATIENTS OVERCOME SKEPTICISM	I BELIEVE PATIENTS WILL HAVE NO OPINION ON THE USE OF AI IN MY PRACTICE	I BELIEVE WITH EDUCATIONAL SESSIONS, WE CAN GET PATIENTS EXCITED ABOUT AI IN PATHOLOGY	I BELIEVE THAT PATIENTS ARE ALREADY AWARE AND EXCITED ABOUT AI IN PATHOLOGY	TOTAL	WEIGHTED AVERAGE
(no label)	3.58%<br>17	9.68%<br>46	50.95%<br>242	29.05%<br>138	6.74%<br>32	475	3.26

Q29 Do you believe that quality assurance can be improved by incorporating AI into your practice?

Answered: 474 Skipped: 6

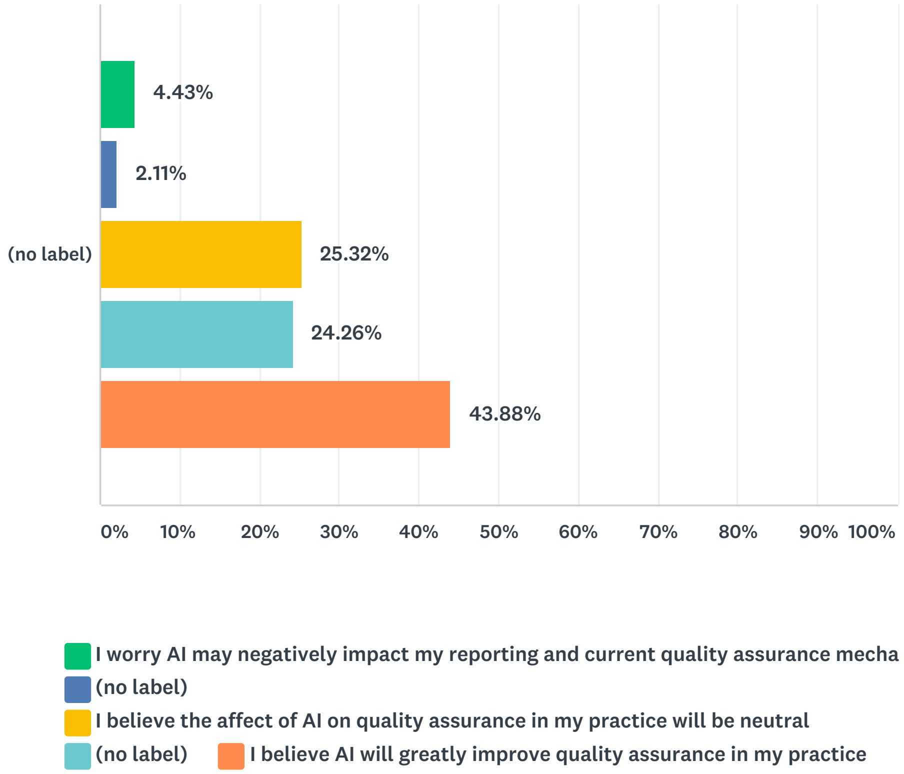

	I WORRY AI MAY NEGATIVELY IMPACT MY REPORTING AND CURRENT QUALITY ASSURANCE MECHANISMS	(NO LABEL)	I BELIEVE THE AFFECT OF AI ON QUALITY ASSURANCE IN MY PRACTICE WILL BE NEUTRAL	(NO LABEL)	I BELIEVE AI WILL GREATLY IMPROVE QUALITY ASSURANCE IN MY PRACTICE	TOTAL	WEIGHTED AVERAGE
(no label)	4.43%<br>21	2.11%<br>10	25.32%<br>120	24.26%<br>115	43.88%<br>208	474	4.01

Q30 If proven useful as a decision support tool, what do you believe should be the balance between pathologist based slide interpretation and AI slide interpretation?

Answered: 471 Skipped: 9

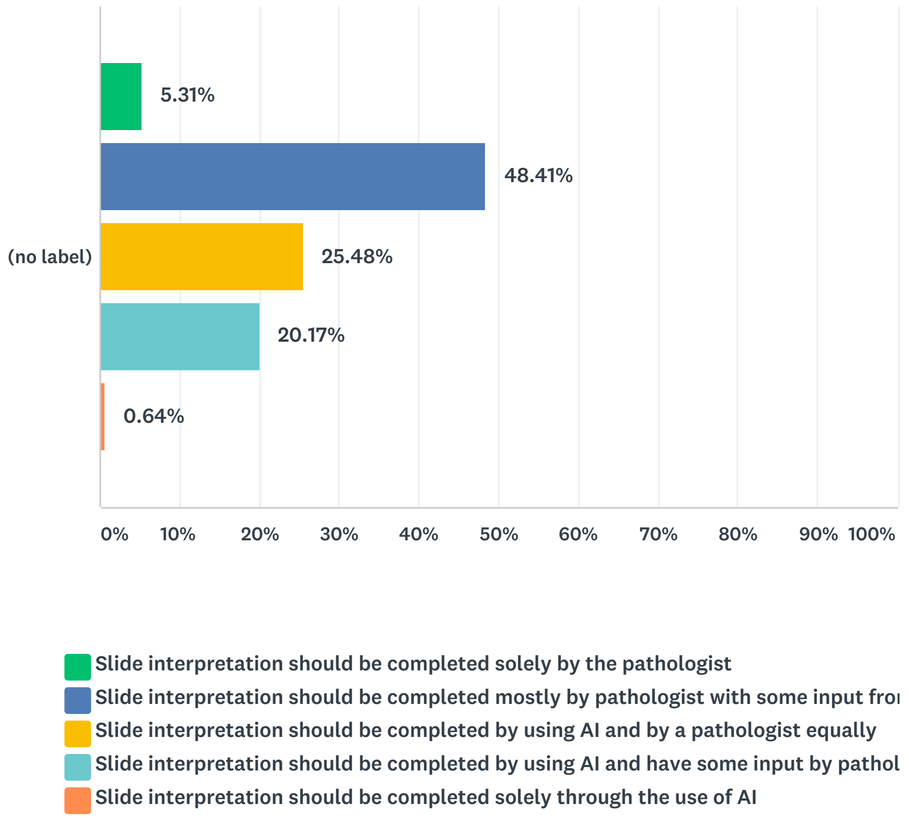

	SLIDE INTERPRETATION SHOULD BE COMPLETED SOLELY BY THE PATHOLOGIST	SLIDE INTERPRETATION SHOULD BE COMPLETED MOSTLY BY PATHOLOGIST WITH SOME INPUT FROM AI	SLIDE INTERPRETATION SHOULD BE COMPLETED BY USING AI AND BY A PATHOLOGIST EQUALLY	SLIDE INTERPRETATION SHOULD BE COMPLETED BY USING AI AND HAVE SOME INPUT BY PATHOLOGIST	SLIDE INTERPRETATION SHOULD BE COMPLETED SOLELY THROUGH THE USE OF AI	TOTAL	WEIGHTED AVERAGE
(no label)	5.31%<br>25	48.41%<br>228	25.48%<br>120	20.17%<br>95	0.64%<br>3	471	2.62

Q31 If AI tools eventually reach the performance of a sub-specialized pathologist, how concerned will you be of diagnostic errors in a setting where AI provides diagnostic reports on their own?

Answered: 473 Skipped: 7

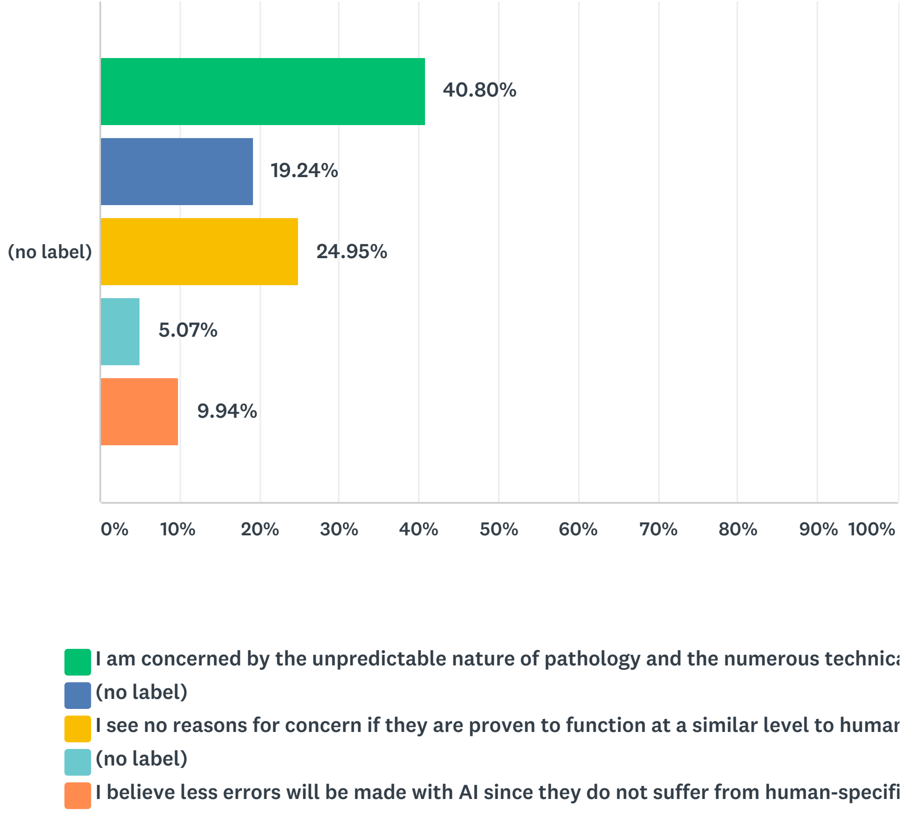

	I AM CONCERNED BY THE UNPREDICTABLE NATURE OF PATHOLOGY AND THE NUMEROUS TECHNICAL ARTIFACTS	(NO LABEL)	I SEE NO REASONS FOR CONCERN IF THEY ARE PROVEN TO FUNCTION AT A SIMILAR LEVEL TO HUMAN PATHOLOGISTS	(NO LABEL)	I BELIEVE LESS ERRORS WILL BE MADE WITH AI SINCE THEY DO NOT SUFFER FROM HUMAN-SPECIFIC ERRORS	TOTAL	WEIGHTED AVERAGE
(no label)	40.80%<br>193	19.24%<br>91	24.95%<br>118	5.07%<br>24	9.94%<br>47	473	2.24

Q32 If AI tools eventually reach the performance of a sub-specialized pathologist, how concerned will you be of diagnostic errors in a setting where AI is used as a decision support tool to help human pathologists provide diagnostic reports?

Answered: 476 Skipped: 4

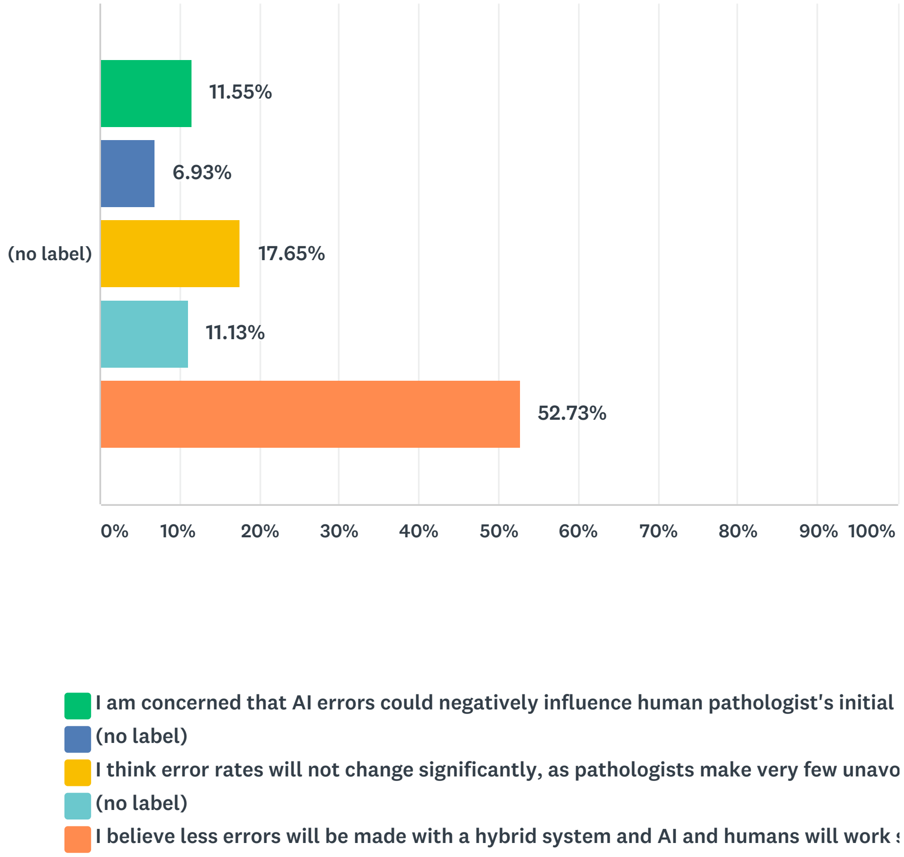

	I AM CONCERNED THAT AI ERRORS COULD NEGATIVELY INFLUENCE HUMAN PATHOLOGIST'S INITIAL INTERPRETATION	(NO LABEL)	I THINK ERROR RATES WILL NOT CHANGE SIGNIFICANTLY, AS PATHOLOGISTS MAKE VERY FEW UNAVOIDABLE ERRORS	(NO LABEL)	I BELIEVE LESS ERRORS WILL BE MADE WITH A HYBRID SYSTEM AND AI AND HUMANS WILL WORK SYNERGISTICALLY	TOTAL	WEIGHTED AVERAGE
(no label)	11.55%<br>55	6.93%<br>33	17.65%<br>84	11.13%<br>53	52.73%<br>251	476	3.87

Q33 If medical error occurs in a setting a where pathologists use AI as a decision support tool, who do you believe should be held legally liable?

Answered: 471 Skipped: 9

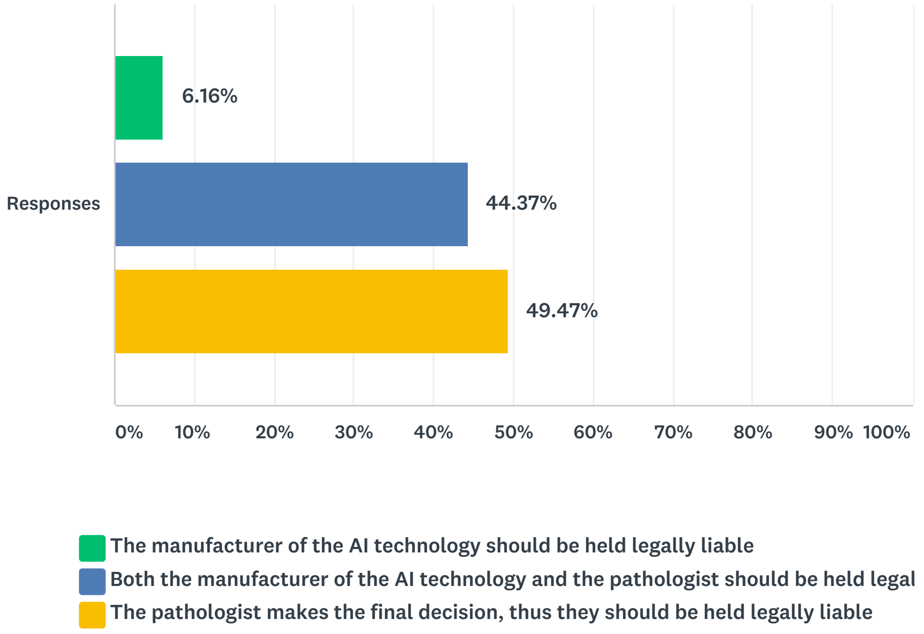

	THE MANUFACTURER OF THE AI TECHNOLOGY SHOULD BE HELD LEGALLY LIABLE	BOTH THE MANUFACTURER OF THE AI TECHNOLOGY AND THE PATHOLOGIST SHOULD BE HELD LEGALLY LIABLE	THE PATHOLOGIST MAKES THE FINAL DECISION, THUS THEY SHOULD BE HELD LEGALLY LIABLE	TOTAL	WEIGHTED AVERAGE
Responses	6.16%<br>29	44.37%<br>209	49.47%<br>233	471	2.43

Q34 Do you believe the routine use of AI in pathologic diagnosis will erode "traditional" skills from practicing pathologists or residents?

Answered: 475 Skipped: 5

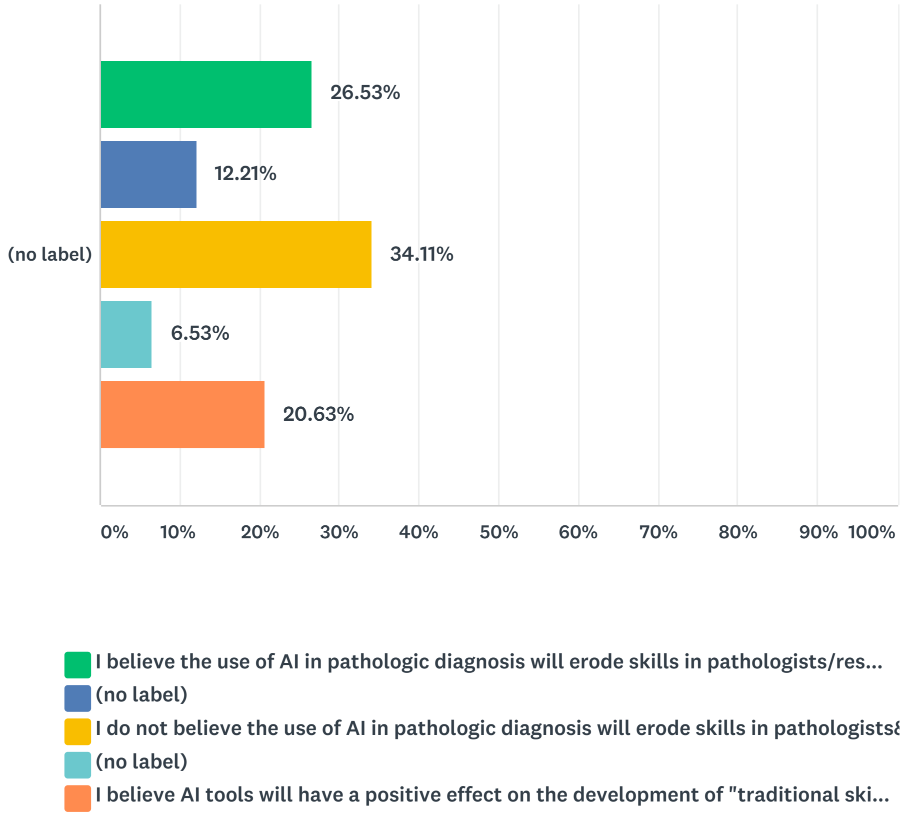

	I BELIEVE THE USE OF AI IN PATHOLOGIC DIAGNOSIS WILL ERODE SKILLS IN PATHOLOGISTS/RESIDENTS	(NO LABEL)	I DO NOT BELIEVE THE USE OF AI IN PATHOLOGIC DIAGNOSIS WILL ERODE SKILLS IN PATHOLOGISTS/RESIDENTS	(NO LABEL)	I BELIEVE AI TOOLS WILL HAVE A POSITIVE EFFECT ON THE DEVELOPMENT OF "TRADITIONAL SKILLS"	TOTAL	WEIGHTED AVERAGE
(no label)	26.53%<br>126	12.21%<br>58	34.11%<br>162	6.53%<br>31	20.63%<br>98	475	2.83

Q35 Do you see your roles and respect from other clinicians changing with the introduction of AI into your practice?

Answered: 475 Skipped: 5

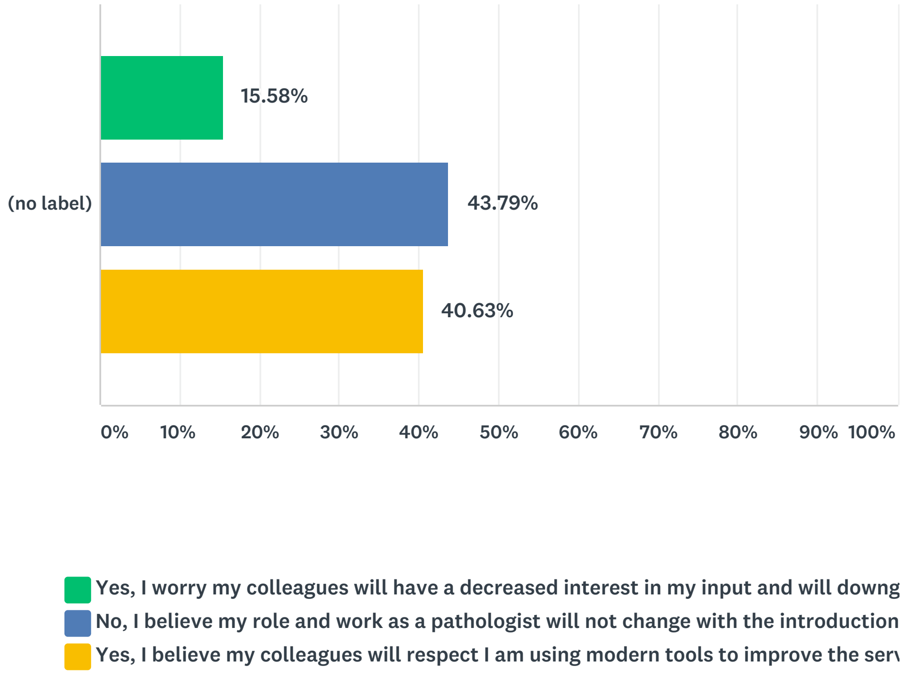

	YES, I WORRY MY COLLEAGUES WILL HAVE A DECREASED INTEREST IN MY INPUT AND WILL DOWNGRADE MY VALUE	NO, I BELIEVE MY ROLE AND WORK AS A PATHOLOGIST WILL NOT CHANGE WITH THE INTRODUCTION OF AI	YES, I BELIEVE MY COLLEAGUES WILL RESPECT I AM USING MODERN TOOLS TO IMPROVE THE SERVICES I PROVIDE	TOTAL	WEIGHTED AVERAGE
(no label)	15.58%<br>74	43.79%<br>208	40.63%<br>193	475	2.25

Q36 How do you see AI affecting pathology training? (Select all that apply)

Answered: 474 Skipped: 6

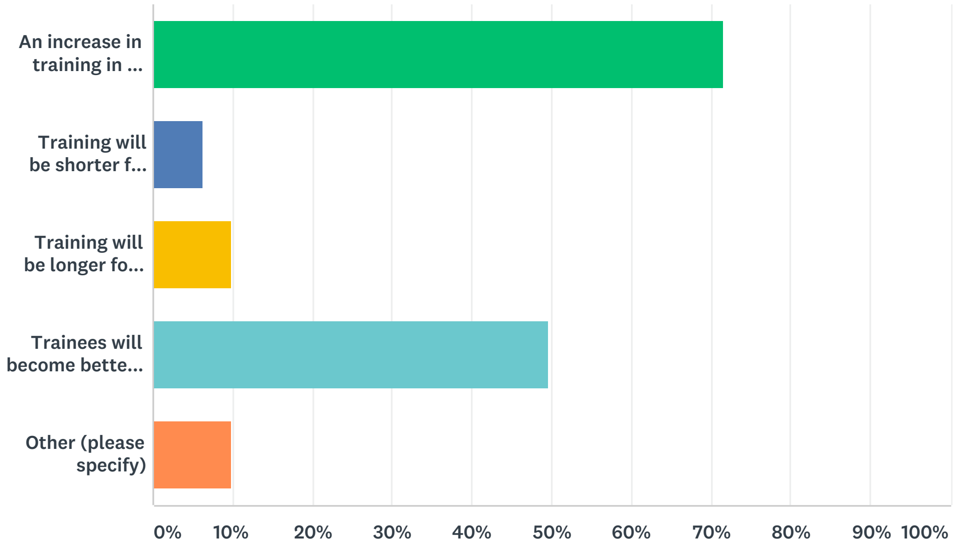

ANSWER CHOICES	RESPONSES	
An increase in training in the understanding and use of informatics in clinical practice during residency/fellowship	71.52%	339
Training will be shorter for pathology residents/fellows	6.33%	30
Training will be longer for pathology residents/fellows	9.92%	47
Trainees will become better at recognizing pathologies with the use of AI	49.58%	235
Other (please specify)	9.92%	47
Total Respondents: 474		

Q37 Do you believe pathologist compensation will be affected with the incorporation of AI into pathology practice?

Answered: 476 Skipped: 4

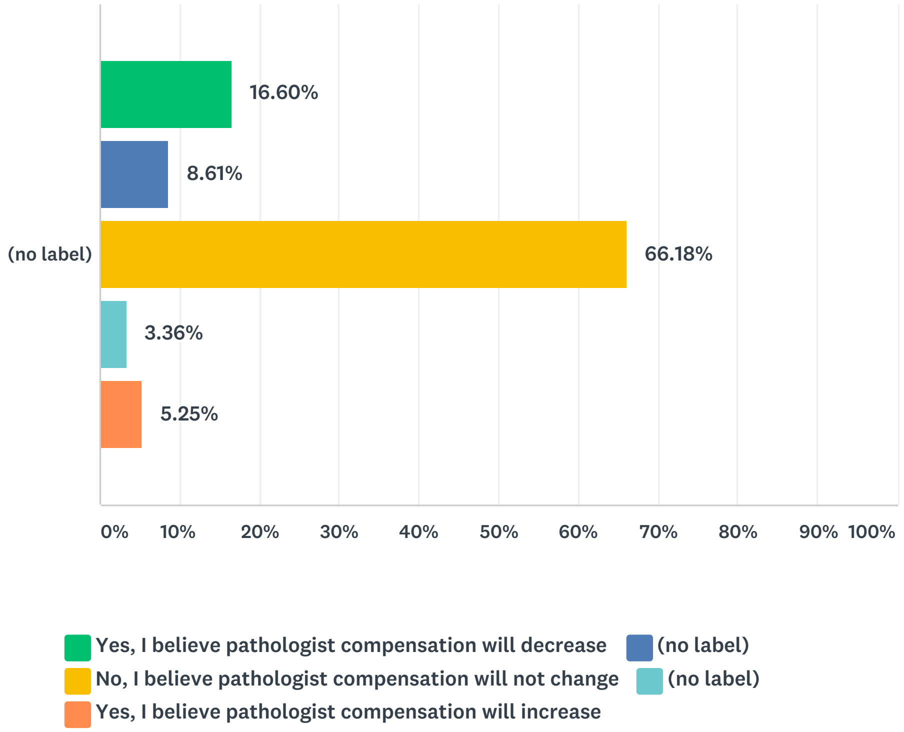

	YES, I BELIEVE PATHOLOGIST COMPENSATION WILL DECREASE	(NO LABEL)	NO, I BELIEVE PATHOLOGIST COMPENSATION WILL NOT CHANGE	(NO LABEL)	YES, I BELIEVE PATHOLOGIST COMPENSATION WILL INCREASE	TOTAL	WEIGHTED AVERAGE
(no label)	16.60% 79	8.61% 41	66.18% 315	3.36% 16	5.25% 25	476	2.72

Q38 Do you believe AI in pathology practice will be used differently in the community compared to an academic setting?

Answered: 475 Skipped: 5

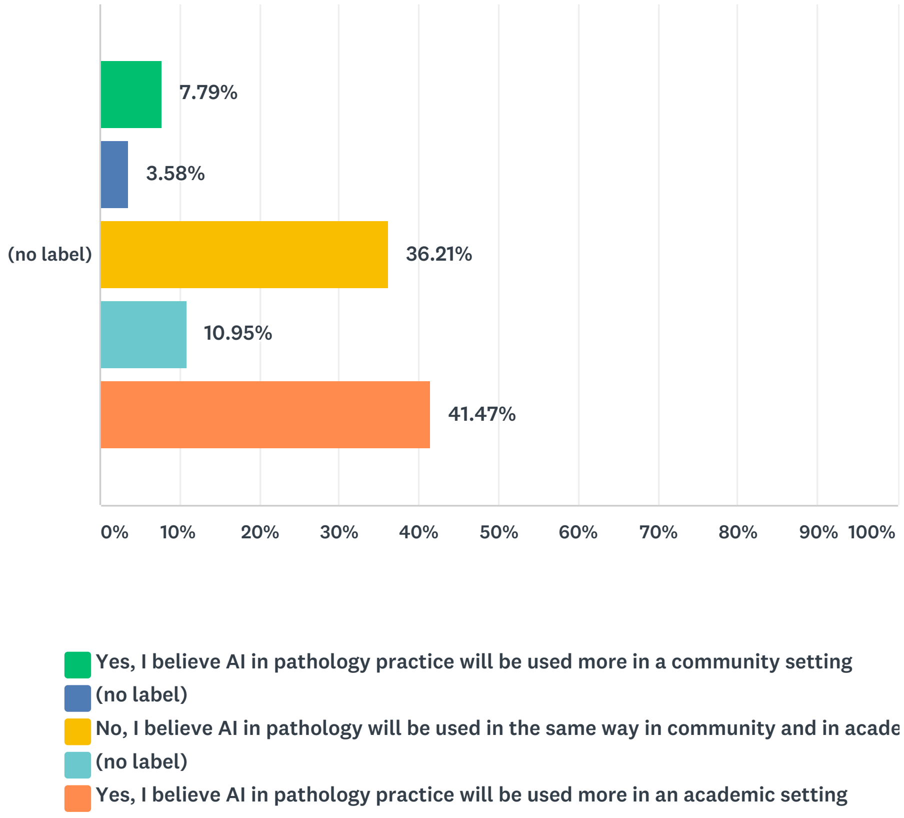

	YES, I BELIEVE AI IN PATHOLOGY PRACTICE WILL BE USED MORE IN A COMMUNITY SETTING	(NO LABEL)	NO, I BELIEVE AI IN PATHOLOGY WILL BE USED IN THE SAME WAY IN COMMUNITY AND IN ACADEMIC SETTINGS	(NO LABEL)	YES, I BELIEVE AI IN PATHOLOGY PRACTICE WILL BE USED MORE IN AN ACADEMIC SETTING	TOTAL	WEIGHTED AVERAGE
(no label)	7.79%<br>37	3.58%<br>17	36.21%<br>172	10.95%<br>52	41.47%<br>197	475	3.75

Q39 How do you believe AI will affect your teaching responsibilities?

Answered: 474 Skipped: 6

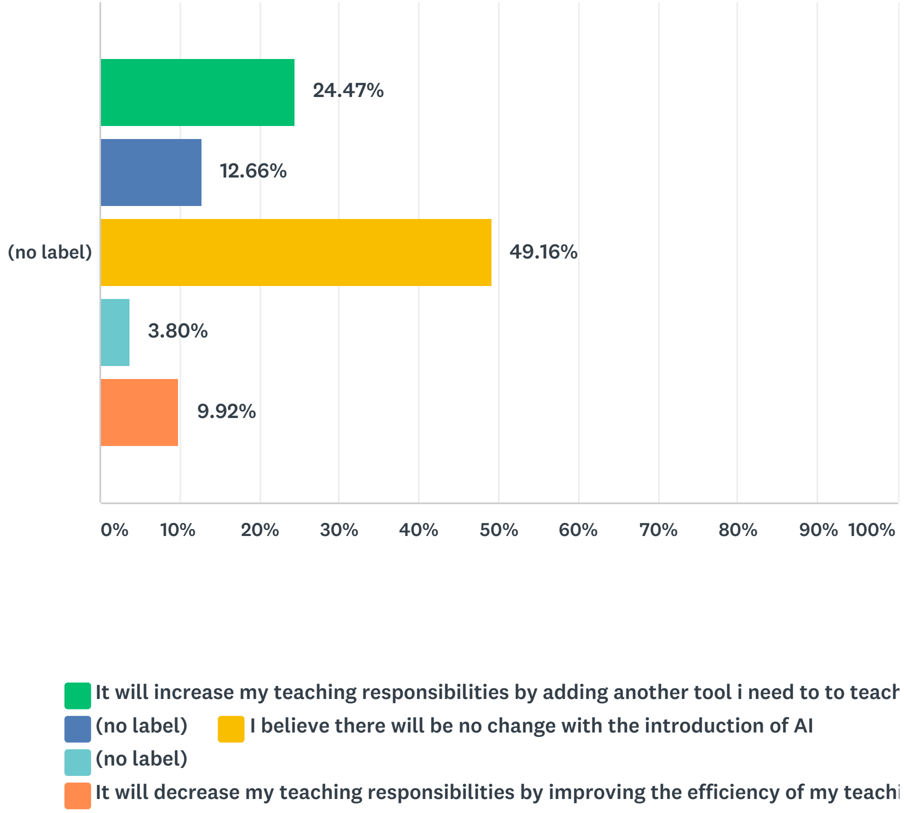

	IT WILL INCREASE MY TEACHING RESPONSIBILITIES BY ADDING ANOTHER TOOL I NEED TO TO TEACH MY RESIDENTS	(NO LABEL)	I BELIEVE THERE WILL BE NO CHANGE WITH THE INTRODUCTION OF AI	(NO LABEL)	IT WILL DECREASE MY TEACHING RESPONSIBILITIES BY IMPROVING THE EFFICIENCY OF MY TEACHING	TOTAL	WEIGHTED AVERAGE
(no label)	24.47%<br>116	12.66%<br>60	49.16%<br>233	3.80%<br>18	9.92%<br>47	474	2.62

Q40 How do you believe AI will affect your research productivity?

Answered: 476 Skipped: 4

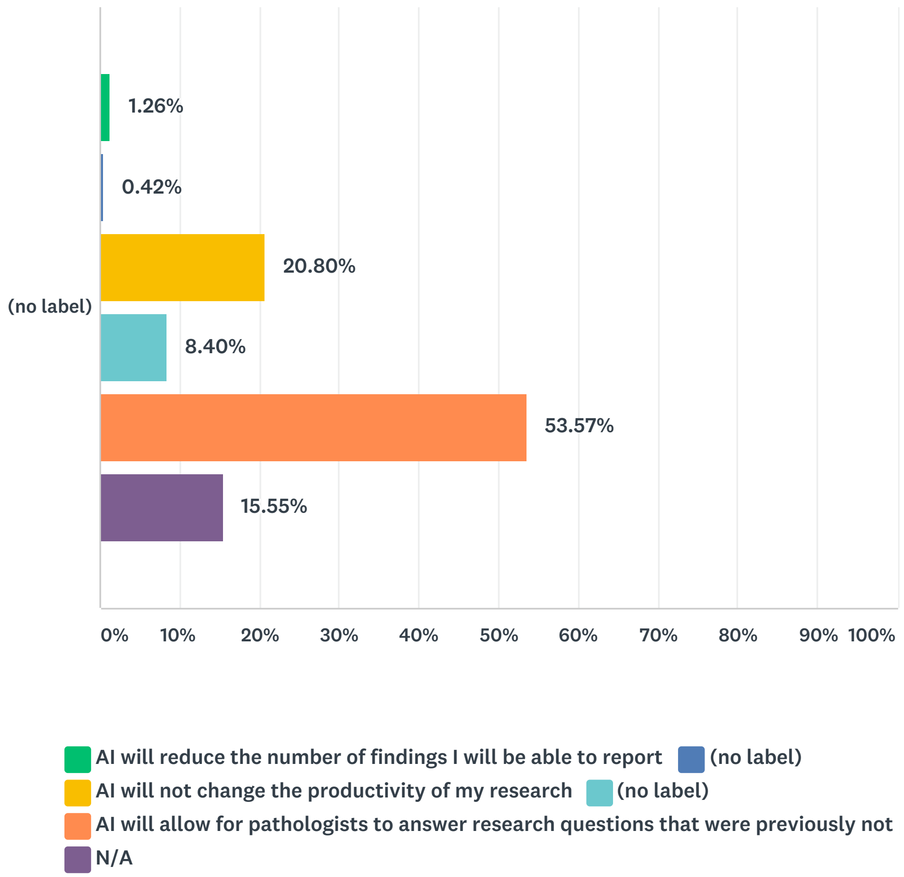

	AI WILL REDUCE THE NUMBER OF FINDINGS I WILL BE ABLE TO REPORT	(NO LABEL)	AI WILL NOT CHANGE THE PRODUCTIVITY OF MY RESEARCH	(NO LABEL)	AI WILL ALLOW FOR PATHOLOGISTS TO ANSWER RESEARCH QUESTIONS THAT WERE PREVIOUSLY NOT POSSIBLE	N/A	TOTAL	WEIGHTED AVERAGE
(no label)	1.26%<br>6	0.42%<br>2	20.80%<br>99	8.40%<br>40	53.57%<br>255	15.55%<br>74	476	4.33

Q41 I believe AI will be incorporated into my practice within:

Answered: 473 Skipped: 7

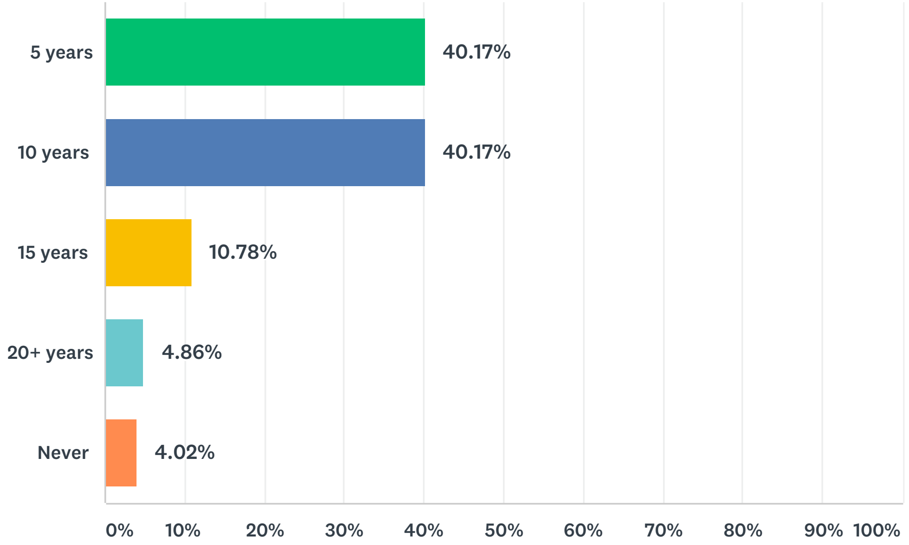

ANSWER CHOICES	RESPONSES	
5 years	40.17%	190
10 years	40.17%	190
15 years	10.78%	51
20+ years	4.86%	23
Never	4.02%	19
TOTAL		473

Q42 I believe AI will be a major and/or routine tool in pathology practice within:

Answered: 476 Skipped: 4

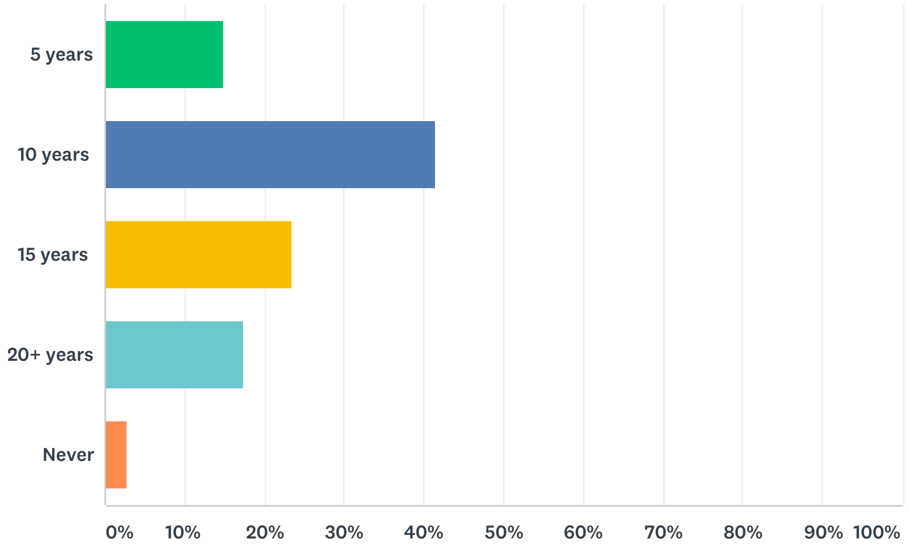

ANSWER CHOICES	RESPONSES	
5 years	14.92%	71
10 years	41.39%	197
15 years	23.53%	112
20+ years	17.44%	83
Never	2.73%	13
TOTAL		476

Q43 Please provide any additional information or comments you may have regarding the use of AI in pathology or this survey (optional).

Answered: 115 Skipped: 365

Supplementary File 2 – Survey Thematic Analysis

Survey Thematic Analysis

Cautious optimism (5%)
- AI has great potential, but it will drastically change the nature of pathology.

AI is inevitable (10%)

AI is coming, but not in the near future (9%)
- AI is inevitable but it will take time before it can fully be incorporated into pathology.

AI will be applicable in certain domains, but not in others (3%)
- More applicable to certain subspecialties within pathology than others.
- Will be more applicable in academic centers than community centers, largely due to financial constraints.

AI will be used as a tool to help pathologists, not replace them (17%)
- Pathologists and AI will have a synergetic relationship.
- AI will complement the current practices of pathologists.
- Pathologist positions will not decrease.

AI will cause the nature of pathology to evolve (9%)
- Pathologists that refuse to adopt AI will be replaced by pathologists that will.
- AI has the potential to be used to share cases among pathologists.

AI will increase precision and accuracy in pathology (5%)
- AI will provide an additional tool for error-prevention.

AI will need to be legally and professionally monitored (8%)
- This may limit the potential of AI.
- AI will require proper testing and trials before implementation.

Digital image analysis will present barriers for AI (3%)
- AI will not be successful while slides are still being used.

AI will increase efficiency in pathology (10%)
- AI will improve efficiency by replacing mundane tasks that pathologists currently dedicate time.

Potential of AI will be demonstrated by how it is accepted in other fields (2%)
- AI is already used in radiology, and will be an indicator of how successful it could be in pathology.

Concerns regarding decrease in pathology job market (3%)
- AI will replace pathologists.

AI will cause changes in pathology training (7%)
- Concerns that incoming pathologists will use AI as a short cut.
- Integration of AI would require new positions for AI specialists.
- AI will cause training

Misconceptions regarding AI must be addressed before it is implemented (5%)
- Require educational sessions.

Skepticism regarding an increase in errors (4%)
- Will AI be able to distinguish between artifacts and mitoses.

Concerns regarding budgetary constraints (4%)

- Incorporation of AI will be limited by funding provided by the government.
- All responses containing this theme were received from residents of the UK.

Comment/concern regarding survey (3%)

- Concerns regarding the formatting of specific questions.
- Respondents expressing interest in topic and survey results.

Uncertainty regarding effects of integrating AI into pathology (2%)
